# Identifying and exploring biohydrogenating rumen bacteria with emphasis on pathways including *trans*-10 intermediates

**DOI:** 10.1186/s12866-020-01876-7

**Published:** 2020-07-07

**Authors:** Lore Dewanckele, Jeyamalar Jeyanathan, Bruno Vlaeminck, Veerle Fievez

**Affiliations:** 1 Laboratory for Animal Nutrition and Animal Product Quality (LANUPRO), Department of Animal Sciences and Aquatic Ecology, Ghent University, Ghent, Belgium; 2 Present address: Research Group Marine Biology, Department of Biology, Ghent University, Ghent, Belgium

**Keywords:** Biohydrogenation, *Cuitbacterium acnes*, Pure cultures, Rumen, *trans*-11 to *trans*-10 shift

## Abstract

**Background:**

Bacteria involved in ruminal formation of *trans*-10 intermediates are unclear. Therefore, this study aimed at identifying rumen bacteria that produce *trans*-10 intermediates from 18-carbon unsaturated fatty acids.

**Results:**

Pure cultures of 28 rumen bacterial species were incubated individually in the presence of 40 μg/mL 18:3*n*-3, 18:2*n*-6 or *trans*-11 18:1 under control or lactate-enriched (200 mM Na lactate) conditions for 24 h. Of the 28 strains, *Cutibacterium acnes* (formerly *Propionibacterium acnes*) was the only bacterium found to produce *trans*-10 intermediates from 18:3*n*-3 and 18:2*n*-6, irrespective of the growth condition. To further assess the potential importance of this species in the *trans*-11 to *trans*-10 shift, different biomass ratios of *Butyrivibrio fibrisolvens* (as a *trans*-11 producer) and *C. acnes* were incubated in different growth media (control, low pH and 22:6*n*-3 enriched media) containing 40 μg/mL 18:2*n*-6. Under control conditions, a *trans*-10 shift, defined in the current study as *trans*-10/*trans*-11 ≥ 0.9, occurred when the biomass of *C. acnes* represented between 90 and 98% of the inoculum. A low pH or addition of 22:6*n*-3 inhibited *cis*-9, *trans*-11 CLA and *trans*-10, *cis*-12 CLA formation by *B. fibrisolvens* and *C. acnes*, respectively, whereby *C. acnes* seemed to be more tolerant. This resulted in a decreased biomass of *C. acnes* required at inoculation to induce a *trans*-10 shift to 50% (low pH) and 90% (22:6*n*-3 addition).

**Conclusions:**

Among the bacterial species studied**,***C. acnes* was the only bacterium that have the metabolic ability to produce *trans*-10 intermediates from 18:3*n*-3 and 18:2*n*-6. Nevertheless, this experiment revealed that it is unlikely that *C. acnes* is the only or predominant species involved in the *trans*-11 to *trans*-10 shift in vivo.

## Background

Ruminant diets commonly contain forages and concentrates, with mainly 18-carbon unsaturated fatty acids (FA) (i.e. linolenic acid, 18:3*n*-3 and linoleic acid, 18:2*n*-6) [1]. Following ingestion, dietary lipids are hydrolyzed and the non-esterified FA are released into the rumen. Unsaturated FA are then converted to saturated FA by rumen bacteria via a process called biohydrogenation [2, 3]. This involves several consecutive conversions via various pathways, resulting in the formation of a plethora of biohydrogenation intermediates, and ultimately in the formation of 18:0 [3]. The predominant biohydrogenation pathways of 18:3*n*-3 and 18:2*n*-6 involve the formation of intermediates containing a double bond in the *trans* configuration at the 11th carbon atom from the carboxyl end, further referred to as *trans*-11 intermediates (i.e. *cis*-9, *trans*-11, *cis*-15 conjugated linolenic acid (CLnA), *trans*-11, *cis*-15 18:2, *cis*-9, *trans*-11 conjugated linoleic acid (CLA) and *trans*-11 18:1) [3]. Nevertheless, under certain dietary conditions (i.e. high starch/low fiber diets or diets supplemented with marine oils or vegetable oils), a shift might occur in biohydrogenation pathway toward the formation of *trans*-10 intermediates (i.e. *trans*-10, *cis*-12, *cis*-15 CLnA, *trans*-10, *cis*-15 18:2, *trans*-10, *cis*-12 CLA and *trans*-10 18:1) at the expense of *trans*-11 intermediates [1, 4, 5], referred to as the *trans*-11 to *trans*-10 shift.

The formation in the rumen of various biohydrogenation intermediates seems the responsibility of several bacterial species (e.g. [6, 7]). Although there might be other bacteria producing *trans*-11 intermediates, some bacteria involved in the *trans*-11 biohydrogenation pathway have been identified. *Butyrivibrio fibrisolvens* was the first ruminal bacterial species found to carry out biohydrogenation of 18:2*n*-6 and 18:3*n*-3 in vitro through the *trans*-11 pathway [6]. Nevertheless, further research identified other *Butyrivibrio* spp., *Pseudobutyrivibrio* spp. and *Sharpea* spp. as also capable of producing *trans*-11 intermediates (e.g. [8–10]). Several other bacterial species can also convert 18:2*n*-6 to *cis*-9, *trans*-11 CLA, e.g. species belonging to the genera *Bifidobacterium* [11], *Lactobacillus* [12], and *Roseburia* [13]. However, all members of the (*Pseudo*) *butyrivibrio* group, including *B. fibrisolvens* and *B. proteoclasticus,* and *Sharpea* spp. convert 18:2*n*-6 much more rapidly than other species [10, 14].

*Trans*-10 intermediates are particularly observed under specific dietary conditions in vivo (i.e. high starch/low fiber diets and supplementation of marine oils or vegetable oils), which are often associated with milk fat depression [4, 15]. Despite the practical and economic relevance of the latter, the rumen bacteria responsible for the formation of *trans*-10 intermediates have not yet been unambiguously identified. Wallace et al. [16] suggested that *Cutibactrium acnes* (formerly *Propionibacterium acnes* [17]) is responsible for the formation of *trans*-10, *cis*-12 CLA from 18:2*n*-6. However, the relevance of this species in inducing a *trans*-10 shift in the rumen is questionable given its very low ruminal abundance [14, 18]. Considering its increasing ruminal abundance in situations with milk fat depression [19–21], *Megasphaera elsdenii* was proposed as an alternative candidate responsible for ruminal *trans*-10, *cis*-12 CLA formation. However, conflicting results upon incubation with 18:2*n*-6 [8, 22] question its role in this process. Kemp et al. [23] observed formation of *trans*-10 18:1 from 18:2*n*-6 and 18:3*n*-3 by *Ruminococcus albus*. Nevertheless, no other studies reported on the 18:2*n*-6 and 18:3*n*-3 metabolism by *R. albus* since 1975 and therefore, its role in ruminal *trans*-10 formation is unclear. Therefore, further research is needed to gain more information about the biohydrogenation ability and pathway of those bacteria.

Correlation analysis based on ruminal bacterial populations and milk [21, 24], blood [17] or rumen FA profiles [17, 25] revealed the possible contribution of several bacterial genera in ruminal formation of *trans*-10 intermediates, i.e. *Acidaminococcus* spp., *Bifidobacterium* spp., *Carnobacterium* spp., *Dialister* spp., and *Lactobacillus* spp. However, in vitro studies using pure cultures are needed to ascertain the capacity of those bacteria to produce *trans*-10 intermediates from 18:3*n*-3 or 18:2*n*-6. Next to this, as *Streptococcus* spp. and *Selenomonas* spp. often increase upon feeding high-grain diets [26, 27], which are often associated with a *trans*-11 to *trans*-10 shift [4]. Investigating the biohydrogenating ability of these bacteria could elucidate their potential involvement in ruminal *trans*-10 formation.

The majority of the bacteria potentially involved in ruminal *trans*-10 formation are related to ruminal lactate metabolism, which is often altered when feeding high-grain diets [28]. As such, lactate might influence the ruminal biohydrogenation process by affecting the metabolism of certain bacteria. Another potential route of *trans*-10 formation is via the formation of hydroxy FA. According to Devillard et al. [29], hydroxy FA produced by *Roseburia* spp. are converted to CLA by a mixed microbial community originating from human feces. As such, 18:3*n*-3 or 18:2*n*-6 might be converted to hydroxy FA by certain rumen species (e.g. *S. bovis* [8]), which might then be converted to *trans*-10 intermediates by other bacteria. Finally, *trans*-10 18:1 might, at least in part, originate from *trans*-11 18:1 in the rumen as Laverroux et al. [30] observed isomerization of *trans*-11 18:1 to *trans*-10 18:1 by mixed cultures in vitro.

The aim of this study was to identify rumen bacteria that produce *trans*-10 intermediates from 18:3*n*-3 or 18:2*n*-6, or their biohydrogenation and biohydrating intermediates. It was hypothesized that at least some of the investigated strains produce *trans*-10 intermediates. Additionally, the effect of supplementation of lactate to the medium on the metabolism of 18:2*n*-6 was investigated. It was hypothesized that lactate-utilizing bacteria would grow better under lactate-enriched conditions and would alter their metabolism and convert 18:2*n*-6 to *trans*-10 intermediates. Finally, the potential importance of *trans*-10 producing bacteria was investigated by using different biomass ratios of bacteria capable of producing *cis*-9, *trans*-11 CLA (*B. fibrisolvens*) and *trans*-10, *cis*-12 CLA (*C. acnes*) from 18:2*n*-6 under different in vitro conditions.

## Results

### Metabolism of linolenic, linoleic, and vaccenic acid by individual species of rumen bacteria (Exp. 1)

The metabolism of 18:3*n*-3, 18:2*n*-6 and *trans*-11 18:1 by the 28 individual strains is presented in Table 1. Nine strains metabolized 18:3*n*-3 and 18:2*n*-6 during a 24-h incubation period. *Butyrivibrio fibrisolvens* D1 showed an accumulation of *trans*-11 intermediates (i.e. *cis*-9, *trans*-11, *cis*-15 CLnA, *trans*-11, *cis*-15 18:2 and *trans*-11 18:1), when incubated with 18:3*n*-3 or 18:2*n*-6. When incubated with 18:2*n*-6, *B. proteoclasticus* P18 additionally produced 18:0 besides *trans*-11 18:1. In contrast to *B. fibrisolvens*, there was only 6% of *trans*-11 (*trans*-11, *cis*-15 18:2) intermediates after 24 h of incubation of *B. proteoclasticus* with 18:3*n*-3. Interestingly, *C. acnes* DSM 1897 was the only strain found to produce *trans*-10, *cis*-12, *cis*-15 CLnA and *trans*-10, *cis*-12 CLA from 18:3*n*-3 and 18:2*n*-6, respectively. However, *trans*-10, *cis*-12, *cis*-15 CLnA represented only 5% of the products formed from 18:3*n*-3. When incubated with 18:3*n*-3, *C. acnes* additionally produced ∆11,13,15–18:3, which represented 50% of the products formed. The final products of *Bifidobacterium adolescentis* RU 424, *Bifidobacterium pseudolongum* RU224, *Streptococcus gallolyticus* DSM 16831, *Streptococcus equinus* Pearl 11, and *M. elsdenii* 2602A and 5052B were mainly hydroxy FA, with the hydroxyl group located at the 10th (i.e. *B. adolescentis*, *B. pseudolongum* and *M. elsdenii* 5052B) and/or at the 13th (i.e. *S. equinus*, *S. gallolyticus*, and *M. elsdenii* 2602A and 5052B) carbon atom from the carboxyl end. *Trans*-11 18:1 was only converted by *B. proteoclasticus* P18 to 18:0. After 24 h of incubation, 51.0 ± 9.52% (mean ± SD) of the initial *trans*-11 18:1 was metabolized to 18:0. No other bacteria metabolized *trans*-11 18:1 (data not shown).

**Table 1:** Metabolism of 18:3*n*-3 and 18:2*n*-6^a^ by different bacterial strains during 24 h of incubation under control growth conditions (Exp. 1). Blank cells related to 18:3*n*-3 and 18:2*n*-6 indicate no metabolism takes place by these strains

Strain	Total VFA formed (μmol/tube; mean ± SD)^b^	VFA products^c^	18:3*n*-3		18:2*n*-6	
			% metabolized (mean ± SD)^d^	Products formed (% of total intermediates formed and remaining 18:3*n*-3)^e,f^	% metabolized (mean ± SD)^d^	Products formed (% of total intermediates and remaining 18:2*n*-6)^e,f^
*Acidaminococcus fermentans* VR4	54 ± 9.5	A, B				
*Acidaminococcus intestini* ADV 255.99	55 ± 13.0	A, B				
*Bifidobacterium adolescentis* RU 424	251 ± 5.4	A	79.8 ± 13.94	10-OH ∆12,15–18:2 (45%)	76.8 ± 6.86	10-OH ∆12–18:1 (62%)
*Bifidobacterium pseudolongum* RU224	210 ± 18.5	A	26.7 ± 14.27	10-OH ∆12,15–18:2 (13%)	23.1 ± 8.56	10-OH ∆12–18:1 (15%)
*Butyrivibrio fibrisolvens* D1	102 ± 45.9	B, A	99.3 ± 0.43	*c*9,*t*11,*c*15 CLnA (48%)	98.0 ± 0.40	*t*11 18:1 (89%)
				*t*11,*c*15 18:2 (43%)		
				t11 C18:1 (6%)		
*Butyrivibrio proteoclasticus* P18	169 ± 12.7	B, A	98.9 ± 0.03	*c*9/*t*13/*t*14 18:1^g^ (42%)	98.4 ± 0.48	18:0 (76%)
				*t*15/*c*11 18:1^g^ (21%)		*t*11 18:1 (17%)
				*c*15 18:1 (17%)		
				18:0 (6%)		
				t11, c15 18:2 (6%)		
*Lactobacillus ruminis* RF1	30 ± 17.2	A, P				
*Lactobacillus ruminis* RF2	14 ± 11.9	A, P				
*Cutibacterium acnes* DSM 1897	99 ± 43.7	P, A	86.5 ± 15.83	∆11,13,15–18:3 (50%)	88.4 ± 7.16	*t*10,*c*12 CLA (75%)
				*t*10,*c*12,*c*15 CLnA (5%)		10-OH 12–18:1 (7%)
*Ruminococcus albus* 7	24 ± 8.6	A				
*Streptococcus equinus* Pearl 11	18 ± 5.2	A	21.6 ± 10.58	13-OH ∆9,15–18:2 (6%)	84.0 ± 2.95	13-OH ∆9–18:1 (69%)
*Streptococcus gallolyticus* DSM 16831	16 ± 5.1	A	96.3 ± 1.49	13-OH ∆9,15–18:2 (86%)	89.8 ± 2.99	13-OH ∆9–18:1 (47%)
						∆9,14–18:2 (32%)
*Megasphaera elsdenii* B159	147 ± 5.6	B				
*Megasphaera elsdenii* T81	126 ± 6.1	B				
*Megasphaera elsdenii* LC1	121 ± 11.2	B, A				
*Megasphaera elsdenii* 2602A	191 ± 21.2	B, P	32.7 ± 8.92	13-OH ∆9,15–18:2 (19%)	81.8 ± 3.71	13-OH ∆9–18:1 (63%)
						∆9,14–18:2 (5%)
*Megasphaera elsdenii* 3016B	138 ± 10.5	B				
*Megasphaera elsdenii* 3218A	134 ± 7.9	B				
*Megasphaera elsdenii* 3436A	117 ± 4.3	B				
*Megasphaera elsdenii* 4251	125 ± 6.8	B				
*Megasphaera elsdenii* 4257	124 ± 5.3	B				
*Megasphaera elsdenii* 4296	120 ± 5.5	B				
*Megasphaera elsdenii* 4400	58 ± 25.8	A, P				
*Megasphaera elsdenii* 5045	128 ± 6.6	B				
*Megasphaera elsdenii* 5052B	63 ± 9.9	A	60.9 ± 11.21	10-OH ∆12,15–18:2 (34%)	88.0 ± 2.20	13-OH ∆9–18:1 (42%)
						10-OH ∆12–18:1 (24%)
*Megasphaera elsdenii* 5596	127 ± 3.1	B				
*Selenomonas ruminantium* GA-192	83 ± 12.0	P, A				
*Selenomonas ruminantium* PC 18	241 ± 31.8	P, A				

^a^ The initial amount of fatty acid was 40 μg/mL

^b^ Measured fermentation products were acetate, propionate, isobutyrate, butyrate, isovalerate, valerate and caproate

^c^ Main VFA product: A, acetate; B, butyrate; P, propionate; in decreasing order of importance. Lactate concentration was not measured

^d^ % metabolized, proportion of the initial 18:3*n*-3 or 18:2*n*-6 which was converted after 24 h of incubation

^e^ Only the intermediates representing ≥5% are presented as its proportion of the sum of total intermediates and remaining initial 18:3*n*-3 or 18:2*n*-6 after 24 h of incubation

^f^*c*, *cis*; *t*, *trans*; CLA, conjugated linoleic acid; CLnA, conjugated linolenic acid. For each of the formed intermediates, the proportion of the respective intermediate on the sum of total produced intermediates and remainder of the initial product (i.e. 18:3*n*-3 or 18:2*n*-6) after 24 h was calculated

^g^ The different isomers could not be separated from each other with the used technique

### Influence of lactate on metabolism of linoleic acid by individual species of rumen bacteria (Exp. 2)

Supplementation of lactate to the growth medium only affected the metabolism of 18:2*n*-6 by two of the studied strains, i.e. *M. elsdenii* 5052B and *B. pseudolongum* RU224 (Table 2). With *M. elsdenii* 5052B, supplementation of lactate to the medium decreased both the OD value (*P* = 0.013; Table S1) as well as the metabolized proportion of 18:2*n*-6 after 24 h of incubation (from 88.01 to 74.35%; *P* = 0.006, Table 2). With *B. pseudolongum* RU224, supplementation of lactate increased the metabolized proportion of 18:2*n*-6 after 24 h of incubation (from 23.06 to 38.41%; *P* = 0.033, Table 2). However, with both bacterial strains, the accumulated intermediates remained the same as under control growth conditions. Although differences were also observed for several other strains in OD value (Table S1) and total VFA produced (Table S2) after 24 h of incubation between the control treatment and the lactate-enriched medium, particularly for the lactate-utilizing bacteria *M. elsdenii* and *Selenomonas ruminantium*, no differences in metabolized proportion of 18:2*n*-6 or in accumulated intermediates were observed for any of the other strains (Table 2).

**Table 2:** Influence of lactate on metabolism of 18:2*n*-6^a^ by different bacterial strains during 24 h of incubation (Exp. 2)

Strain^b^	Control		Lactate		SEM	*P*-value
	% metabolized^c^	Main products formed (% of total intermediates and remaining 18:2*n*-6)^d,e^	% metabolized^c^	Main products formed (% of total intermediates and remaining 18:2*n*-6)^d,e^		
*Bifidobacterium adolescentis* RU 424	76.75	10-OH ∆12–18:1 (62%)	82.22	10-OH ∆12–18:1 (67%)	2.859	0.234
*Bifidobacterium pseudolongum* RU224	23.06	10-OH ∆12–18:1 (15%)	38.41	10-OH ∆12–18:1 (18%)	3.716	0.033
*Butyrivibrio fibrisolvens* D1	98.00	*t*11 18:1 (89%)	97.90	*t*11 18:1 (90%)	0.217	0.765
*Butyrivibrio proteoclasticus* P18	98.35	18:0 (69%)	98.54	18:0 (76%)	0.248	0.295
		*t*11 18:1 (22%)		*t*11 18:1 (17%)		
*Cutibacterium acnes* DSM 1897	88.41	***t*10**,*c*12 CLA (75%)	60.79	***t*10**,*c*12 CLA (39%)	20.522	0.395
		10-OH 12–18:1 (7%)		10-OH 12–18:1 (5%)		
*Streptococcus equinus* Pearl 11	83.96	13-OH ∆9–18:1 (69%)	80.92	13-OH ∆9–18:1 (64%)	1.493	0.223
*Streptococcus gallolyticus* DSM 16831	89.75	13-OH ∆9–18:1 (47%)	92.77	∆9,14–18:2 (56%)	1.877	0.319
		∆9,14–18:2 (32%)		13-OH ∆9–18:1 (27%)		
*Megasphaera elsdenii* 2602A	81.82	13-OH ∆9–18:1 (63%)	79.13	13-OH ∆9–18:1 (50%)	2.287	0.452
		∆9,14–18:2 (5%)		∆9,14–18:2 (10%)		
				c9, t13, t14 18:1 (7%)		
*Megasphaera elsdenii* 5052B	88.01	13-OH ∆9–18:1 (42%)	74.35	13-OH ∆9–18:1 (34%)	1.786	0.006
		10-OH ∆12–18:1 (24%)		10-OH ∆12–18:1 (16%)		
				c9, t13, t14 18:1 (5%)		

^a^ The initial amount of 18:2*n*-6 was 40 μg/mL

^b^ Only the strains which metabolized 18:2*n*-6 are shown

^c^ % metabolized, proportion of the initial 18:2*n*-6 which was converted after 24 h of incubation

^d^ Only the intermediates representing ≥5% of the sum of total intermediates and remaining initial 18:2*n*-6 after 24 h of incubation are presented in this table

^e^*c*, *cis*; *t*, *trans*; CLA, conjugated linoleic acid. For each of the formed intermediates, the proportion of the respective intermediate on the sum of total produced intermediates and remainder of 18:2*n*-6 after 24 h was calculated

### Effect of growth medium on metabolism of 18:2*n*-6 by monocultures of *B. fibrisolvens* D1 and *C. acnes* DSM 1897 (Exp. 3)

**Fig. 1:**
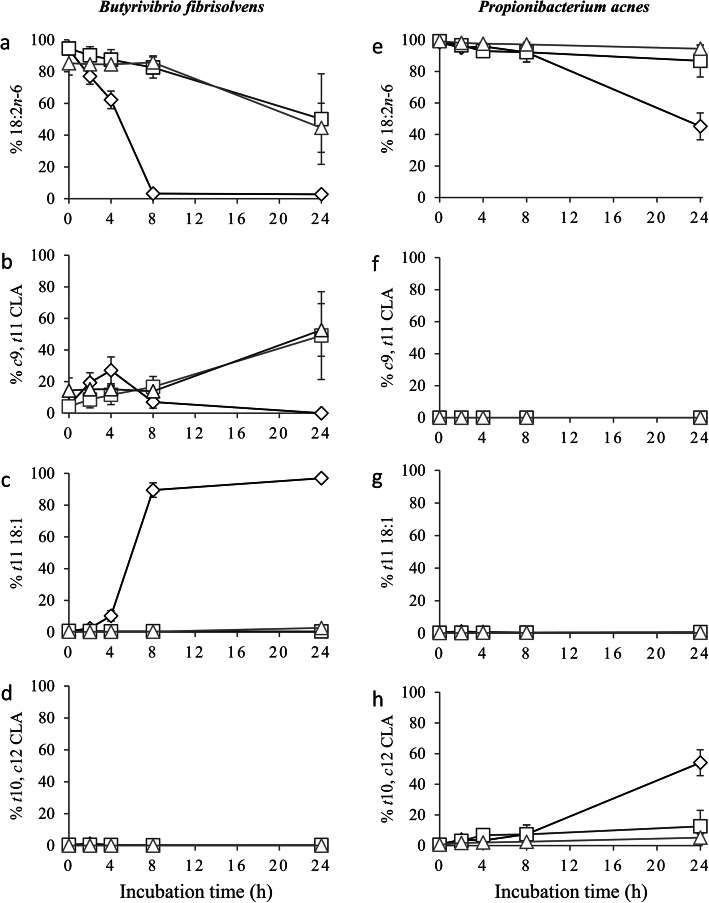
Effect of growth medium on the proportion of 18:2*n*-6 and its biohydrogenation intermediates (% of total intermediates and 18:2*n*-6) during a 24 h incubation period with *Butyrivibrio fibrisolvens* D1 (left; **a**–**d**) and *Cutibacterium acnes* DSM 1897 (right; **e**–**h**) (Exp. 3). The initial concentration of 18:2*n*-6 was 40 μg/mL. Control medium (diamond); low pH medium (square), control medium with pH adjusted to 5.5; DHA-enriched (docosahexaenoic acid) medium (triangle), control medium containing 40 μg/mL of 22:6*n*-3. *c*, *cis*; *t*, *trans*; CLA, conjugated linoleic acid

The growth medium generally affected the metabolism of 18:2*n*-6 during the 24 h incubation period, the mean proportions of 18:2*n*-6, *cis*-9, *trans*-11 CLA, *trans*-11 18:1, and *trans*-10, *cis*-12 CLA differed between bacterial species (*P*-values for interaction effect ranging from < 0.001 to 0.011; Table 3). With *B. fibrisolvens*, 18:2*n*-6 was almost completely metabolized after 8 h of incubation under control growth conditions (Fig. 1a). This disappearance of 18:2*n*-6 was accompanied by a transient accumulation of *cis*-9, *trans*-11 CLA (Fig. 1b), which was further transformed to *trans*-11 18:1 (Fig. 1c). No *trans*-10, *cis*-12 CLA (Fig. 1d) or 18:0 (data not shown) was formed by *B. fibrisolvens*. The average 18:2*n*-6 proportion was higher (*P* <  0.001) with the low pH and DHA-enriched media compared with the control medium (Table 3, Fig. 1a), which implies inhibition of the formation of *cis*-9, *trans*-11 CLA (Fig. 1b). These conditions also inhibited further transformation of *cis*-9, *trans*-11 CLA to *trans*-11 18:1, as the proportion of *trans*-11 18:1 was lower (*P* <  0.001) with the low pH and DHA-enriched media compared with the control medium (Table 3, Fig. 1c).

**Table 3:** Average proportions^a^ (% of total intermediates and 18:2*n*-6) of 18:2*n*-6 and its biohydrogenation intermediates over a 24 h incubation period under different growth conditions^b^ with mono-cultures of *Butyrivibrio fibrisolvens* D1 or *Propionibacterium acnes* DSM 1897 (Exp. 3)

	*B. fibrisolvens*			*C. acnes*			SEM	*P*-value		
	Control	Low pH	DHA	Control	Low pH	DHA		Bacterium	Growth medium	Bacterium × growth medium
18:2*n*-6	20.72^β^*	73.86^α^*	71.49^α^*	77.00^β^	86.88^α^	95.21^α^	3.918	< 0.001	< 0.001	< 0.001
*cis*-9, *trans*-11 CLA^c^	8.01^β^	25.49^α^*	27.24^α^*	0.54	0.41	0.54	3.273	< 0.001	0.013	0.011
*trans*-11 18:1	71.10^α^*	0.55^β^	1.08^β^	0.64	4.46	0.33	1.959	< 0.001	< 0.001	< 0.001
*trans*-10, *cis*-12 CLA^c^	< 0.01*	< 0.01*	0.40	21.81^α^	8.25^β^	3.76^β^	2.073	< 0.001	0.001	< 0.001

^a^ The average proportions of FA over the 24 h incubation period were computed as the area under the curve divided by the total duration of incubation (24 h), using the individual measured proportions for each FA at the different sampling times

^b^ Low pH, control medium with pH adjusted to 5.5; DHA (docosahexaenoic acid), control medium containing 40 μg/mL of 22:6*n*-3; all growth media contained 40 μg/mL of 18:2*n*-6

^c^ CLA, conjugated linoleic acid

^α, β^ Means differ (*P* <  0.05) between growth media within the same bacterial species

* Means differ (*P* <  0.05) between *B. fibrisolvens* and *C. acnes* within the same growth medium

Under control growth conditions, *C. acnes* converted 18:2*n*-6 to *trans*-10, *cis*-12 CLA, but this conversion to CLA was more slowly compared to *B. fibrisolvens* (Table 3, Fig. 1a and e). A low pH or addition of DHA to the medium increased the mean proportion of residual 18:2*n*-6 (*P* <  0.001; Table 3, Fig. 1e), which was accompanied with a decreased proportion of *trans*-10, *cis*-12 CLA (*P* <  0.001; Table 3, Fig. 1h). This implies inhibition of the formation of *trans*-10, *cis*-12 CLA by low pH and by DHA. This inhibition of 18:2*n*-6 isomerization was less pronounced with *C. acnes* compared with *B. fibrisolvens*. There was no formation of *cis*-9, *trans*-11 CLA (Fig. 1f), *trans*-11 18:1 (Fig. 1g), *trans*-10 18:1 (data not shown), or 18:0 (data not shown) by *C. acnes*.

### Effect of ratio of *B. fibrisolvens* D1 to *C. acnes* DSM 1897 in the inoculum in combination with varying growth media on the *trans*-11 to *trans*-10 shift and volatile fatty acid production (Exp. 4)

**Fig. 2:**
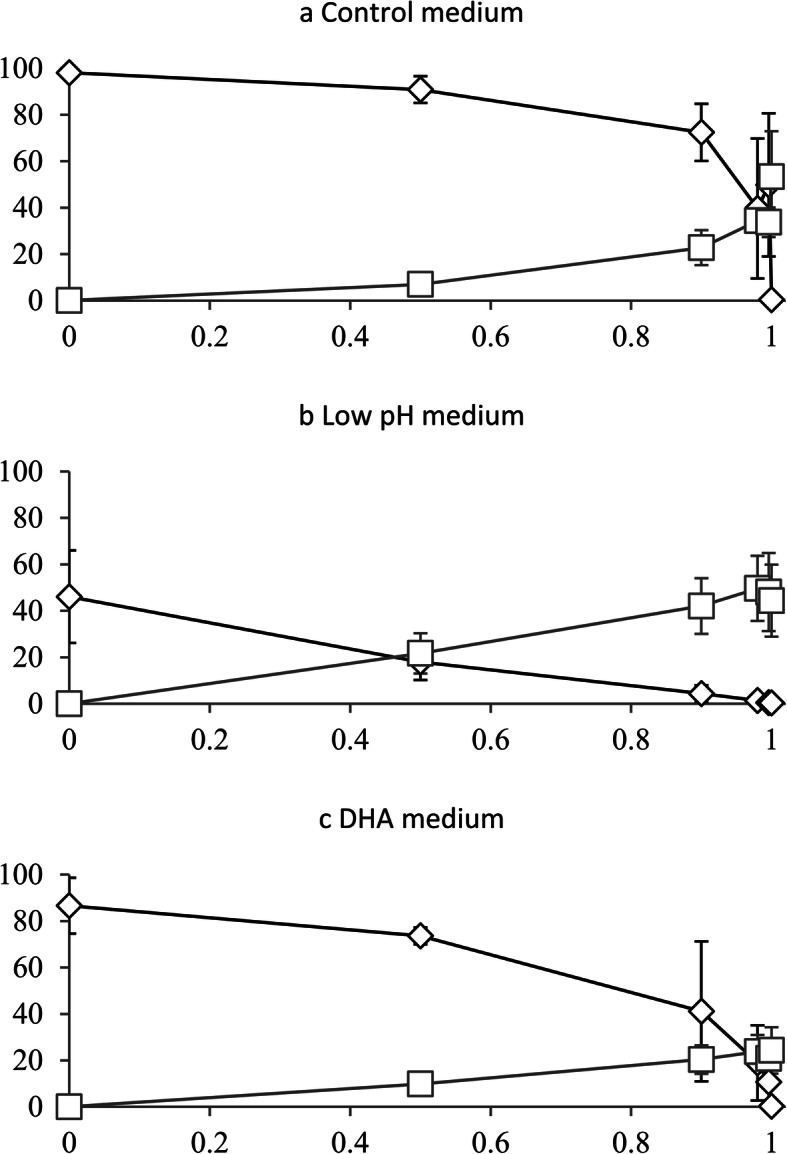
Proportion (% of total intermediates and 18:2*n*-6) of *cis*-9, *trans*-11 CLA + *trans*-11 18:1 (diamond) and *trans*-10, *cis*-12 CLA + *trans*-10 18:1 (square) after a 24 h incubation period in relation to the relative biomass of *Cutibacterium acnes* DSM 1897 in the inoculum. The initial concentration of 18:2*n*-6 was 40 μg/mL. **a**, control medium; **b**, low pH medium, control medium with pH adjusted to 5.5; **c**, DHA-enriched (docosahexaenoic acid) medium, control medium containing 40 μg/mL of 22:6*n*-3

The biomass ratio of *B. fibrisolvens* to *C. acnes* in the inoculum had an effect on the disappearance of 18:2*n*-6 (Table 4; *P* <  0.05). The mean proportion of 18:2*n*-6 over the 24 h incubation period increased with increasing biomass amounts of *C. acnes* in the inoculum (*P* <  0.001). Increasing biomass of *C. acnes* also increased the accumulation of *trans*-10, *cis*-12 CLA (*P* ≤ 0.007) at the expense of *cis*-9, *trans*-11 CLA (*P* = 0.013), with a *trans*-11 to *trans*-10 shift, defined in the current experiment as *trans*-10/*trans*-11 ≥ 0.9, occurring when the relative biomass of *C. acnes* in the inoculum was between 90 and 98% (Table 4, Fig. 2a). In mono-cultures of *C. acnes* (Exp. 3 and Exp. 4), metabolism of 18:2*n*-6 resulted in the accumulation of *trans*-10, *cis*-12 CLA, whereas in co-culture with *B. fibrisolvens* (Exp. 4), *trans*-10, *cis*-12 CLA was partially further metabolized to *trans*-10 18:1 (Table 4), indicating that the latter bacterium is responsible for the conversion of *trans*-10, *cis*-12 CLA to *trans*-10 18:1.

**Table 4:** Average proportions^a^ (% of total intermediates and 18:2*n*-6) of 18:2*n*-6^b^ and its biohydrogenation intermediates over a 24 h incubation period with different ratios of *Butyrivibrio fibrisolvens* D1 to *Propionibacterium acnes* DSM 1897 in the inoculum under control growth conditions (Exp. 4)

	*B. fibrisolvens* (%)/C*. acnes* (%)						SEM	*P*-value	
	100/0	50/50	10/90	2/98	0.4/99.6	0/100		Linear	Quadratic
18:2*n*-6	12.35	19.59	34.90	49.57	53.30	66.65	12.928	< 0.001	< 0.001
*cis*-9, *trans*-11 CLA^c^	19.22	22.44	10.57	8.89	4.15	0.02	7.797	0.013	0.090
*trans*-11 18:1	67.98	51.72	34.59	14.22	15.62	0.41	14.947	< 0.001	0.035
*trans*-10, *cis*-12 CLA^c^	< 0.01	3.03	11.55	20.08	19.13	32.92	3.123	< 0.001	0.007
*trans*-10 18:1	0.45	3.22	8.39	7.24	7.80	< 0.01	4.090	0.014	0.471
*trans*-10/*trans*-11^d^	–	0.08	0.48	3.00	4.04	–	4.436	< 0.001	< 0.001

^a^ The average proportions of FA over the 24 h incubation period were computed as the area under the curve divided by the total duration of incubation (24 h), using the individual measured proportions for each FA at the different sampling times

^b^ The initial amount of 18:2*n*-6 was 40 μg/mL

^c^ CLA, conjugated linoleic acid

^d^ Ratio of *trans*-10 intermediates (*trans*-10, *cis*-12 CLA + *trans*-10 18:1) to *trans*-11 intermediates (*cis*-9, *trans*-11 CLA + *trans*-11 18:1)

-: The ratio is not relevant for mono-cultures as *trans*-11 and *trans*-10 are produced exclusively with mono-cultures of *B. fibrisolvens* and *C. acnes*, respectively

The effect of the ratio of *B. fibrisolvens* to *C. acnes* in the inoculum on 18:2*n*-6 metabolism depended on the growth medium (Table 5; *P* <  0.05). With the low pH medium or the DHA-enriched medium, an increase in residual 18:2*n*-6 after 24 h of incubation was observed compared to the control medium, which implies that a low pH or the addition of DHA to the medium reduced the rate of 18:2*n*-6 disappearance (Table 5). With the DHA-enriched medium, the amount of 18:2*n*-6 after the 24 h incubation period increased with increasing biomass of *C. acnes* in the inoculum (*P* ≤ 0.014), which was also the case under control conditions. In contrast to this, with the low pH medium, the amount of 18:2*n*-6 after 24 h of incubation was relatively constant, irrespective of the bacterial ratio (*P* ≥ 0.05).

**Table 5:** Proportion (% of total intermediates and 18:2*n*-6) of 18:2*n*-6 and its biohydrogenation intermediates after 24 h of incubation under different growth conditions^a^ with different biomass ratios of *Butyrivibrio fibrisolvens* D1 to *Propionibacterium acnes* DSM 1897 in the inoculum (Exp. 4)

		*B. fibrisolvens* (%)/*C. acnes* (%)						SEM	*P*-value		
		100/0	50/50	10/90	2/98	0.4/99.6	0/100		b	Linear^c^	Quadratic^d^
18:2*n*-6	Control	1.81	2.18	4.76	25.95	16.44	46.67	13.050	R***	< 0.001	0.004
	Low pH	53.91*	60.34*	53.52*	48.95*	51.49*	55.24		M***	0.579	0.320
	DHA	13.28	20.29	38.47*	57.28*	64.67*	75.28*		R × M***	< 0.001	0.014
*c*9, *t*11 CLA^e^	Control	0.20	18.28	14.75	7.78	5.03	< 0.01	7.534	R***	< 0.001	0.004
	Low pH	45.51*	17.59	4.03	0.95	< 0.01	< 0.01		M***	< 0.001	0.563
	DHA	82.52*	70.06*	38.85*	18.20	9.79	< 0.01		R × M***	0.828	0.043
*trans*-11 18:1	Control	97.93	72.57	57.69	31.88	44.80	0.46	9.460	R**	0.772	0.977
	Low pH	0.57*	0.38*	0.37*	0.44*	0.42*	0.39		M***	0.988	0.990
	DHA	4.11*	2.15*	2.28*	0.74*	2.36*	0.36		R × M***	< 0.001	0.086
*t*10, *c*12 CLA^f^	Control	< 0.01	2.63	8.72	18.98	13.61	53.37	6.606	R***	< 0.001	0.629
	Low pH	< 0.01	21.70*	42.04*	49.66*	48.01*	44.37		M***	< 0.001	0.767
	DHA	< 0.01	8.15	19.97	23.57	21.69	24.29*		R × M**	< 0.001	0.019
*trans*-10 18:1	Control	0.06	4.34	14.08	15.40	20.11	< 0.01	3.927	R*	0.905	0.943
	Low pH	< 0.01	< 0.01	0.04*	< 0.01*	0.08*	< 0.01		M***	0.992	0.998
	DHA	0.08	< 0.01	0.42*	0.21*	0.90*	0.06		R × M*	< 0.001	0.911
*trans*-10/*trans*-11^g^	Control	–	0.08	0.34	2.46	2.44	–	9.296	R***	0.017	0.082
	Low pH	–	1.22	12.67	65.74*	90.01*	–		M***	< 0.001	< 0.001
	DHA	–	0.10	0.95	2.86	9.74	–		R × M**	< 0.001	0.003

^a^ Low pH, control medium with pH adjusted to 5.5; DHA (docosahexaenoic acid), control medium containing 40 μg/mL of 22:6*n*-3; all growth media contained 40 μg/mL of 18:2*n*-6

^b^ R, effect of ratio of *B. fibrisolvens* to *C. acnes* in the inoculum; M, effect of growth medium; * 0.01 ≤ *P* < 0.05; ** 0.001 ≤ *P* < 0.01; *** *P* < 0.001

^c^ Linear effect of R within each growth medium

^d^ Quadratic effect of R within each growth medium

^e^*c*9, *t*11 CLA, *cis*-9, *trans*-11 conjugated linoleic acid

^f^*t*10, *c*12 CLA, *trans*-10, *cis*-12 conjugated linoleic acid

^g^ Ratio of *trans*-10 intermediates (*trans*-10, *cis*-12 CLA + *trans*-10 18:1) to *trans*-11 intermediates (*cis*-9, *trans*-11 CLA + *trans*-11 18:1)

* Means differ (*P* < 0.05) from the control growth medium within the same ratio

-: The ratio is not relevant for mono-cultures as *trans*-11 and *trans*-10 are produced exclusively with mono-cultures of *B. fibrisolvens* and *C. acnes*, respectively

Under control conditions, a *trans*-11 to *trans*-10 shift (i.e. *trans*-10/*trans*-11 ≥ 0.9) was observed when *C. acnes* represented between 90 and 98% of the inoculum biomass. With the addition of DHA, this shift already occurred at lower biomass proportions of *C. acnes* (i.e. between 50 and 90%; Table 5, Fig. 2c vs. Figure 2a). The biomass proportion of *C. acnes* necessary to induce this shift was further decreased with the low pH medium, in which a relative biomass proportion of 50% *C. acnes* in the inoculum was sufficient to induce a *trans*-11 to *trans*-10 shift (Table 5, Fig. 2b vs. Figure 2a).

**Fig. 3:**
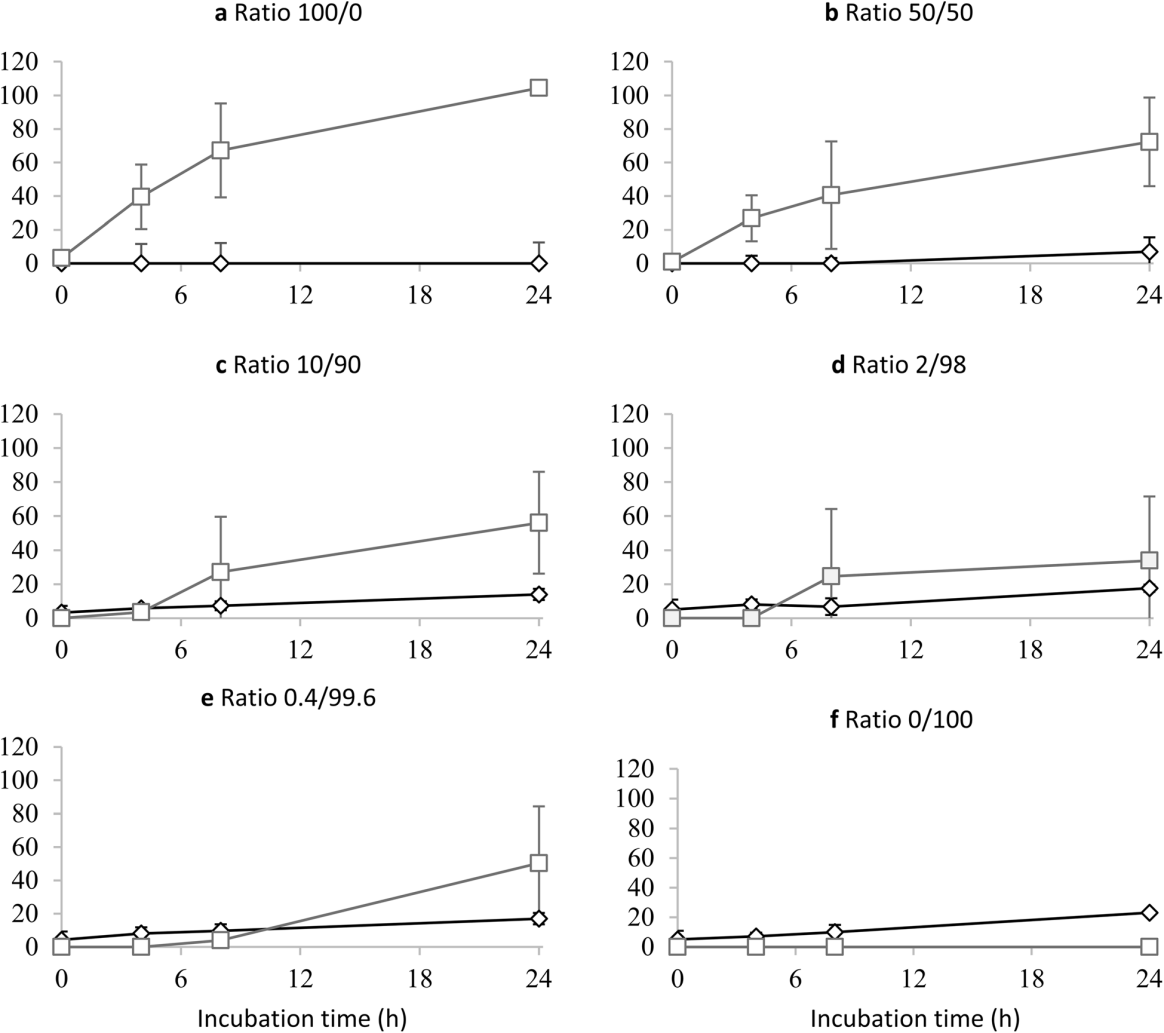
Net production (μmol per tube) of propionic acid (diamond) and butyric acid (square) during a 24 h incubation period with different biomass ratios of *Butyrivibrio fibrisolvens* D1 to *Cuitibacterium acnes* DSM 1897 in the inoculum under control growth conditions

The mono-culture experiment (Exp. 3) revealed that *B. fibrisolvens* primarily produced butyric acid and *C. acnes* primarily generated propionic acid (data not shown). Under control growth conditions in experiment 4, there was a linear increase in propionic acid production (*P* <  0.001) and a linear decrease in butyric acid production (*P* = 0.022) with increasing relative biomass of *C. acnes* in the inoculum (Table S3). Figure 3 shows that there was a delay in butyric acid production under control growth conditions in the first 8 h when the relative biomass of *C. acnes* in the inoculum was 90% or higher (Fig. 3c-e). A low pH or the addition of DHA to the medium reduced the net production of propionic acid and butyric acid by *C. acnes* and *B. fibrisolvens*, respectively (*P* <  0.05; Table S3). The inhibitory effect of low pH on VFA production was higher compared to the addition of DHA. Moreover, the reduction in VFA production due to low pH and DHA addition was more pronounced for butyric acid as compared to propionic acid.

### Metabolism of hydration intermediates by mixed rumen inoculum (Exp. 5)

The hydroxy FA formed by *B. adolescentis* RU 424, *B. pseudolongum* RU224, *S. gallolyticus* DSM 16831, *S. equinus* Pearl 11, and *M. elsdenii* 2602A and 5052B were extensively converted by the mixed rumen community during 24 h of incubation, although in each incubation flask, hydroxy FA remained at the end of the incubation (< 6%, data not shown). Products formed were 18:0 and several unknown FA with a retention time close to that of the initial hydroxy FA. Nevertheless, the mixed rumen inoculum did not produce any *trans*-10 intermediates from the hydroxy FA during 24 h of incubation.

## Discussion

### Metabolism of linolenic, linoleic and vaccenic acid by pure cultures of individual rumen bacteria

In the rumen, 18:3*n*-3 and 18:2*n*-6 are converted to 18:0 via a process called biohydrogenation [3]. Their most common biohydrogenation pathway is via the formation of *trans*-11 intermediates. However, under certain dietary conditions, a shift in biohydrogenation pathway occurs toward the formation of *trans*-10 intermediates at the expense of *trans*-11 intermediates, which is often associated with milk fat depression [4, 5]. Despite the practical and economic relevance of the latter, the rumen bacteria responsible for the formation of *trans*-10 intermediates have not yet been unambiguously identified. Therefore, 28 different rumen species were incubated individually in the presence of 40 μg/mL 18:3*n*-3 or 18:2*n*-6 (Exp. 1). If available, bacterial strains isolated from the rumen were used, if not, strains originated from the gut or feces from other animals or from human tissue were used. Since biohydrogenation of polyunsaturated FA is species and strain specific (e.g. [10, 31]), different strains were included for each genus if available. As *Butyrivibrio* spp. are well-known *trans*-11 producers (e.g. [6, 9]), two *Butyrivibrio* species, i.e. *B. fibrisolvens* and *B. proteoclasticus*, were included in this study as a negative control. In accordance with previous reports [12, 32], *B. fibrisolvens* formed *trans*-11 intermediates from 18:3*n*-3 (i.e. *cis*-9, *trans*-11, *cis*-15 CLnA and *trans*-11, *cis*-15 18:2) and 18:2*n*-6 (i.e. *trans*-11 18:1). *B. proteoclasticus* additionally produced 18:0 from 18:2*n*-6 as observed by Maia et al. [8].

Other genera included in experiment 1 were selected based on results from the literature. Previous in vivo experiments in our laboratory [18, 21] revealed the potential contribution of genera *Acidaminococcus*, *Bifidobacterium* and *Lactobacillus* in the ruminal formation of *trans*-10 intermediates. Moreover, previous in vitro experiments with *Lactobacillus* spp., isolated from cheese [12] or from the human intestine [33], showed *trans*-10, *cis*-12 CLA formation from 18:2*n*-6 by various species of this genus. Nevertheless, from the three genera investigated here, only *Bifidobacterium* metabolized 18:3*n*-3 and 18:2*n*-6 in the current study, and no *trans*-10 intermediates were formed. In contrast to other studies [11, 34, 35], in which formation of *trans*-11 intermediates was observed by *Bifidobacterium* spp., the two *Bifidobacterium* species used in our study produced hydroxy FA after 24 h of incubation.

Originally, four other bacteria (i.e. *Carnobacterium divergens* 66, *Carnobacterium maltaromaticum* MX 5, *Dialister invisus* E7.25 and *Dialister pneumosintes* Cal 4692-1-74) were also included in this study because of positive correlations between their rumen or buccal abundance and *trans*-10 intermediates in the rumen, milk or blood [18, 21, 25]. However, as those bacteria did not grow in our growth medium, which reflects the rumen conditions, it was not possible to investigate their potential metabolism of 18:3*n*-3 and 18:2*n*-6 in the current study. To our knowledge, rumen isolates from the genera *Carnobacterium* and *Dialister* are currently not available. Isolation of those genera from the rumen could thus resolve this issue.

Milk fat depression or situations associated with greater *trans*-10 accumulation are often associated with increased ruminal abundance of *M. elsdenii* [18, 19, 21], *Streptococcus* spp. and *Selenomonas* spp. (e.g. [26, 27]), suggesting that those bacteria are involved in the formation of *trans*-10 intermediates from 18:2*n*-6 or 18:3*n*-3. Indeed, Kim et al. [22] found that two strains of ruminal *M. elsdenii* (i.e. YJ-4 and T81) converted 18:2*n*-6 to *trans*-10, *cis*-12 CLA. In contrast, neither of the two *M. elsdenii* strains analyzed in the study of Maia et al. [8], i.e. LC1 and T81, formed *trans*-10, *cis*-12 CLA. In the current study, fourteen different strains of *M. elsdenii*, including LC1 and T81, were incubated with 18:3*n*-3 and 18:2*n*-6. Under the experimental conditions used here, only two strains were able to metabolize 18:3*n*-3 and 18:2*n*-6, i.e. 2602A and 5052B. Nevertheless, the formed intermediates were mainly hydroxy FA rather than the expected *trans*-10 intermediates. Those results suggest that it is unlikely that *M. elsdenii* directly contributes to the *trans*-11 to *trans*-10 shift. The two *S. ruminantium* strains used in this study did not convert 18:3*n*-3 or 18:2*n*-6. In contrast, both *S. equinus* and *S. gallolyticus* converted 18:3*n*-3 and 18:2*n*-6, but - as for the two *Megasphaera* strains - the accumulated FA were identified as hydroxy FA rather than *trans*-10 intermediates, which is in accordance with Maia et al. [8].

*R. albus* and *C. acnes* were selected based on pure culture studies, which suggested *trans*-10 production by the respective bacteria. Kemp et al. [23] observed formation of *trans*-10 18:1 from 18:2*n*-6 and 18:3*n*-3 by a strictly anaerobic bacterium isolated from sheep rumen, designated as *R. albus*. However, the strain used in the current study did not convert 18:3*n*-3 or 18:2*n*-6. Up till now, no other studies investigated 18:3*n*-3 or 18:2*n*-6 metabolism by this particular species. In accordance with Wallace et al. [16] and McKain et al. [7], *C. acnes* produced *trans*-10, *cis*-12 CLA from 18:2*n*-6. When incubated with 18:3*n*-3, *C. acnes* also formed a *trans*-10 intermediate, i.e. *trans*-10, *cis*-12, *cis*-15 CLnA, which was not observed by Maia et al. [36] using the same strain. The isomerase isolated from *C. acnes* showed the capablilty of converting 18:3*n*-3 to CLnA isomers with *trans*-11,*trans*-13,*cis*-15 CLn as the main product with trace amounts of *trans*-10,*cis*-12,*cis*-15-CLn [37]. In the study of Alves and Bessa [38] *trans*-10, *cis*-15 was observed in rumen contents and they suggested that it may have originated from *trans*-10, *cis*-12, *cis*-15 CLnA. However, in their study, the incubation of 18:3*n*-3 with rumen contents resulted in too limited amounts of ∆10,12,15-CLnA to conclude on possible pathways. Indeed, Zenad et al. [39] also reported similar observation: the *trans-*10 shift when incubating 18:2*n*-6 with ruminal fluids from cows receiving a high-starch plus oil diet or with high starch substrate, while no *trans-*10 shift was observed when 18:3*n*-3 was incubated with this rumen fluid. This suggests that 18:3*n*-3 may be less important precursor of the *trans*-10 shift.

Besides *C. acnes*, none of the investigated bacteria converted 18:2*n*-6 or 18:3*n*-3 to *trans*-10 intermediates. However, some bacteria produced hydroxy FA, i.e. *B. adolescentis*, *B. pseudolongum*, *S. equinus*, *S. gallolyticus*, and *M. elsdenii* 2602A and 5052B. According to Devillard et al. [29], 10-OH cis-12 18:1 might be converted to *cis*-9, *trans*-11 CLA by a mixed community of bacteria originating from the hindgut. As such, we further investigated whether the formed hydration products are converted to *trans*-10 intermediates by mixed rumen inoculum (Exp. 5). Nevertheless, none of the produced hydroxy FA were converted to *trans*-10 intermediates by mixed rumen inoculum after 24 h incubation. The conversion of trans-10 intermediates to 18:0 is slower than the formation of trans-10 intermediates [40]. Therefore, if any trans-10 intermediates were formed, they would have been visible at the end of the incubation period (24 h) as long as potential precursors of trans-10 intermediates are present in the incubation, which was the case here.

Contrasting results between different studies using the same bacterial species or strains might indicate that not only the species or strain itself is important, but that also specific conditions, which are apparently still unknown, are needed to produce *trans*-10 intermediates. Since the majority of the used bacteria are related to ruminal lactate metabolism, which is often altered when feeding high-grain diets [28], we investigated the effect of supplementation of lactate to the medium on the metabolism of 18:2*n*-6 (Exp. 2). It was hypothesized that lactate-utilizing bacteria would grow better under lactate-enriched conditions and would alter their metabolism and convert 18:2*n*-6 to *trans*-10 intermediates. As expected, higher densities and increased accumulation of fermentation products were observed after 24 h of incubation for most strains of the lactate-utilizing bacteria *M. elsdenii* and *S. ruminantium*, which might reflect a better growth of these bacteria under these conditions (Tables S1 and S2). Additionally, the volatile fatty acid profile of *M. elsdenii* changed in lactate-enriched media except for *M. elsdenii* 5045 and *M. elsdenii* 5052B: under control conditions, the primary VFA was butyrate, while in lactate-enriched media the primary VFA product was propionate. Nevertheless, this higher density and fermentation activity were not associated with an altered metabolism of 18:2*n*-6. In contrast, the strain *M. elsdenii* 5052B showed a lower OD value upon incubation with lactate, which was accompanied by a lower proportion of 18:2*n*-6 disappearance. The lactate concentration used in the current experiment (200 mM) was considerably higher than the normal range reported in the rumen (9–30 mM). This potentially impacted the growth of *M. elsdenii* 5052B negatively. Indeed, the rate of lactate utilization and the lactate degradation pathway of *M. elsdenii,* depends on the lactate concentration in the media [41]. However, originally, a lower lactate concentration (i.e. 9 mM) was used (data not shown). As bacterial growth remained unchanged (based on OD600), it was decided to supply lactate according to the growth medium specifications for *M. elsdenii* in Weimer and Moen [42].

Since *trans*-11 18:1 was partly isomerized to *trans*-10 18:1 by mixed cultures in vitro in the study of Laverroux et al. [30], the studied bacteria were additionally incubated in the presence of 40 μg/mL *trans*-11 18:1 (Exp. 1). Nevertheless, none of the studied bacteria isomerized *trans*-11 18:1 to *trans*-10 18:1. *Trans*-11 18:1 was only converted by *B. proteoclasticus* to yield 18:0, in accordance with McKain et al. [7].

In summary, of the 28 studied rumen bacteria, only *C. acnes* was found to have the metabolic ability to produce *trans*-10 intermediates from 18:3*n*-3 or 18:2*n*-6 under the studied circumstances. Nevertheless, the relevance of this species in ruminal *trans*-10 formation is questionable given its very low rumen abundance [14, 18], although bacterial activity and kinetics of its 18:2*n*-6 and 18:3*n*-3 conversion might play a role besides abundance. Therefore, its biohydrogenation of 18:2*n*-6 was further investigated and compared with the well-known *trans*-11 producer *B. fibrisolvens* (Exp. 3 and 4). Furthermore, the effect of different in vitro conditions was investigated (Exp. 3 and 4). Although it could have been of more interest to use a strain originating from the rumen for those experiments, a rumen strain of *C. acnes* is not commercially available and the one isolated by the group of Wallace [16] had been lost (Wallace R. J., personal communication). Nevertheless, in their comparative study [16], similar production rates of *trans*-10, *cis*-12 CLA were observed by *C. acnes* DSM 1897 and *C. acnes* G449, which was isolated from the rumen.

### Assessing the potential importance of *C. acnes* in the *trans*-11 to *trans*-10 shift

*B. fibrisolvens* in mono-culture (Exp. 3) converted 18:2*n*-6 rapidly (i.e. within the first 8 h) to *cis*-9, *trans*-11 CLA, which was then further transformed to *trans*-11 18:1. No *trans*-10, *cis*-12 CLA or 18:0 was formed by this bacterium, which is in accordance with previous reports [7, 13]. The conversion of 18:2*n*-6 to *trans*-10, *cis*-12 CLA by mono-cultures of *C. acnes* (Exp. 3) was five times slower than the conversion by *B. fibrisolvens*, which might be related to the slower growth of this bacterial species under the studied circumstances, as measured by the OD_600_ (data not shown). The lack of *trans*-10 18:1 and 18:0 formation by this bacterium indicates that *trans*-10, *cis*-12 CLA is the end product of 18:2*n*-6 metabolism by *C. acnes*, which is in accordance with a report of McKain et al. [7]. The presence of *trans*-10 18:1 in co-cultures of these bacterial species (Exp. 4) suggests the possibility of *B. fibrisolvens* being able to metabolize *trans*-10, *cis*-12 CLA. Indeed, Kepler et al. [6] and McKain et al. [7] showed that the reductase of pure cultures of *B. fibrisolvens* is not highly specific and can convert *trans*-10, *cis*-12 CLA to *trans*-10 18:1. Nevertheless, the formation rate of *trans*-10 18:1 was lower compared with the formation rate of *trans*-11 18:1, which is also in agreement with the observations of Kepler et al. [6].

As a consequence of the slower conversion of 18:2*n*-6 to *trans*-10, *cis*-12 CLA compared with the conversion to *cis*-9, *trans*-11 CLA, increasing biomass of *C. acnes* in the inoculum in the co-culture experiment (Exp. 4) resulted in an increase in residual 18:2*n*-6. This confirms the results with mono-cultures of *B. fibrisolvens* and *C. acnes* (Exp. 3), which implies that there was no interaction between both bacterial species. The increase in residual 18:2*n*-6 was, however, more pronounced when *C. acnes* was predominantly present in the initial inoculum. This suggests that *B. fibrisolvens* was responsible for much of the disappearance of 18:2*n*-6, even when the latter was not the most abundant bacterium initially present. This can be confirmed as *cis*-9, *trans*-11 CLA, *trans*-11 18:1, and *trans*-10 18:1 production was observed in those co-cultures where *C. acnes* was predominantly present initially. These FA were not detected in mono-cultures of *C. acnes* (Exp. 3), indicating they were produced by *B. fibrisolvens*. Additionally, both propionate and butyrate production was observed in those co-cultures. The production of these metabolites could be used as an indication for the growth or activity of *B. fibrisolvens* and *C. acnes* in the co-cultures of experiment 4, as mono-cultures of these bacterial species (Exp. 3) produced butyric acid and propionic acid, respectively. Even when *B. fibrisolvens* was hardly present initially, this bacterial species started to grow and was getting important after 4 to 8 h as suggested by the increase in butyrate concentrations.

The results of this study show that an increasing relative biomass of *C. acnes* results in an increase in the ratio of *trans*-10 to *trans*-11 intermediates, and that relatively large biomass proportions of *C. acnes* (> 90%) in the inoculum are necessary to induce a *trans*-11 to *trans*-10 shift under control growth conditions investigated here. This might indicate that the *trans*-10 shift, as observed in vivo, is not merely a reflection of the relative amounts of *cis*-9, *trans*-11 CLA and *trans*-10, *cis*-12 CLA producers. Indeed, *B. fibrisolvens* are by far the most abundant biohydrogenating bacteria in the rumen among the cultured bacteria [43], and the relative amount of *C. acnes* is an order of magnitude lower, even under rumen conditions with a *trans*-10/*trans*-11 ratio of 0.82 [14, 22]. Perhaps, *C. acnes* behaves in a different way in the rumen as compared to the in vitro observations in the current experiment. As we observed higher relative proportions of *C. acnes* in the oral cavity compared to the rumen in a previous experiment [21], *trans*-10 formation might perhaps partly take place in the cow’s mouth. Nevertheless, further research is required to confirm this hypothesis. Alternatively, the contribution of *C. acnes* to the *trans*-10 shift observed in the rumen might be limited and other bacterial species could be responsible for the production of *trans*-10, *cis*-12 CLA in vivo, which might be more competitive with *B. fibrisolvens* (higher growth rate, higher rate of CLA formation, …).

Because the *trans*-11 to *trans*-10 shift is described as occurring when rumen pH is low or when marine oils are fed [5], we hypothesized that a low pH or the addition of DHA to the medium would inhibit the formation of *cis*-9, *trans*-11 CLA and/or stimulate the formation of *trans*-10, *cis*-12 CLA. However, in this study, a low pH or the addition of DHA reduced the in vitro disappearance of 18:2*n*-6 by mono-cultures of both *B. fibrisolvens* and *C. acnes* (Exp. 3), but the inhibitory effect of low pH or DHA was smaller for *C. acnes* (14% reduction) compared to *B. fibrisolvens* (52% reduction). Similarly, a low pH or DHA was also more detrimental to butyrate production by *B. fibrisolvens* than to propionate production by *C. acnes*. Indeed, Choi et al. [44] suggested that *trans*-10, *cis*-12 CLA-producing rumen bacteria may be more acid-tolerant than *cis*-9, *trans*-11 CLA-producing rumen bacteria. Probably, they may also be more tolerant to the presence of polyunsaturated FA, in this case DHA. Supportive of this is the fact that *P. freudenreichii* seems to be more tolerant to higher concentrations of 18:2*n*-6 [45] compared to *B. fibrisolvens* [46]. However, this tolerance seems to be strain specific [45]. This higher tolerance of *trans*-10, *cis*-12 CLA producers to polyunsaturated FA was also observed in the experiment of Shingfield et al. [14], in which dietary supplementation of fish oil decreased the relative abundance of *B. fibrisolvens* + *Pseudobutyrivibrio* spp. and tended to increase the amount of *C. acnes* in omasal content of dairy cows.

Although the inhibitory effect of low pH or DHA was smaller for *trans*-10, *cis*-12 CLA compared to *cis*-9, *trans*-11 CLA formation, the absolute amounts of *cis*-9, *trans*-11 CLA formed by mono-cultures of *B. fibrisolvens* were still higher than *trans*-10, *cis*-12 CLA formed by mono-cultures of *C. acnes* (Exp. 3). Nevertheless, a low pH or the addition of DHA to the medium reduced the relative biomass of *C. acnes* needed at inoculation to induce a *trans*-11 to *trans*-10 shift (Exp. 4), with a low pH reducing this biomass more (50%) than DHA addition (90%).

Under the conditions of this study, the results might indicate that diets inducing a low rumen pH may be more provocative for a *trans*-11 to *trans*-10 shift compared to supplementation with marine oils. In agreement with this, Toral et al. [47] observed a *trans*-11 to *trans*-10 shift in the rumen after dietary supplementation of starch and sunflower oil, whereas this shift was not observed after dietary supplementation of fish oil. Similarly, dietary supplementation of different levels of fish oil did not result in a *trans*-11 to *trans*-10 shift in the experiment of Shingfield et al. [14], which was also the case in the experiment of Zhu et al. [48] after dietary supplementation of DHA-enriched microalgae. In contrast to this, dietary supplementation of DHA-enriched microalgae did induce a *trans*-11 to *trans*-10 shift in the experiment of Boeckaert et al. [49]. Probably, the effect of DHA supplementation on rumen biohydrogenation is related to its supplementation level [45], and potentially also to the ruminant species to which marine products are supplemented [50].

Supplementation of marine lipids results in the accumulation of 18:1 isomers in vivo [49, 51], both of *trans*-11 18:1 and *trans*-10 18:1 (e.g. [14, 48, 51]). This accumulation is mostly due to inhibition of the final step of biohydrogenation to 18:0. Inhibition of the transformation of *cis*-9, *trans*-11 CLA to *trans*-11 18:1 by *B. fibrisolvens* upon DHA supplementation to the medium is thus surprising, and might indicate that the contribution of this species to the in vivo formation of *trans*-11 18:1 is less important than originally assumed. Alternatively, *B. fibrisolvens* behaves in a different way in the rumen as compared with the in vitro observations in the current experiment. The media used for the current experiments contained centrifuged rumen fluid which lacked particles. According to Harfoot et al. [52], rumen fluid particles can reduce the toxicity of PUFA and stimulate the biohydrogenation.

As differences in biohydrogenation kinetics between different in vitro conditions depended on bacterial species, the results of this experiment should be taken with caution. The high required biomass proportion of *C. acnes* at inoculation to induce a *trans*-11 to *trans*-10 shift is potentially not only the result of the specific strains which were used, but the specific in vitro conditions could also have strongly influenced this required proportion. Furthermore, the observed strain specificity further complicates extrapolation of our in vitro results to in vivo conditions.

## Conclusions

Among the bacterial species studied, *C. acnes* was the only bacterium having the metabolic ability to produce *trans*-10 intermediates from 18:3*n*-3 and 18:2*n*-6 under the studied circumstances. Nevertheless, it seems unlikely that *C. acnes* is the only or predominant species involved in the *trans*-11 to *trans*-10 shift in vivo. Other bacteria were confirmed or found to produce *trans*-11 intermediates (i.e. *B. fibrisolvens* and *B. proteoclasticus*) or hydroxy FA (i.e. *B. adolescentis*, *B. pseudolongum*, *S. equinus*, *S. gallolyticus*, *M. elsdenii* 2602A and 5052B), which were not further converted to *trans*-10 intermediates by mixed rumen inoculum. None of the studied bacteria isomerized *trans*-11 18:1 to *trans*-10 18:1, and addition of lactate to the medium did not alter the metabolism of the bacteria to produce *trans*-10 intermediates. Nevertheless, the results of this study should be taken with caution as not only bacterial but also environmental features influence competition between bacteria. As such, the in vitro conditions might have strongly affected the obtained results. Furthermore, biohydrogenation seems to be very strain specific, which also complicates extrapolation to in vivo conditions.

## Methods

### Micro-organisms and growth conditions

Batch in vitro incubations were established using pure cultures of 28 rumen bacterial species. The provenance of those species is shown in Table 6. If available, bacterial strains isolated from the rumen were used, if not, strains originated from the gut or feces from other animals or from human were used.

**Table 6:** Provenance of the different bacterial strains used in the experiment

No.	Family	Genus	Species	Strain	Origin	Comments	Source
1	Acidaminococcaceae	*Acidaminococcus*	*fermentans*	VR4	Pig gut, unknown^a^	Type strain	DSMZ
2	Acidaminococcaceae	*Acidaminococcus*	*intestini*	ADV 255.99	Human peritoneal fluid, France	Type strain	DSMZ
3	Bifidobacteriaceae	*Bifidobacterium*	*adolescentis*	RU 424	Bovine rumen, unknown^a^		DSMZ
4	Bifidobacteriaceae	*Bifidobacterium*	*pseudolongum*	RU224	Rumen, unknown^a^	Subsp. *globosum*, type strain	DSMZ
5	Lachnospiraceae	*Butyrivibrio*	*fibrisolvens*	D1	Bovine rumen, unknown^a^	Type strain	DSMZ
6	Lachnospiraceae	*Butyrivibrio*	*proteoclasticus*	P18	Sheep rumen, UK		Dr. J. Wallace
7	Lactobacillaceae	*Lactobacillus*	*ruminis*	RF1	Bovine rumen, unknown^a^	Type strain	DSMZ
8	Lactobacillaceae	*Lactobacillus*	*ruminis*	RF2	Bovine rumen, unknown^a^		DSMZ
9	Propionibacteriaceae	*Propionibacterium*	*acnes*	DSM 1897	Acne lesion in human facial skin, unknown^a^	Type strain	DSMZ
10	Ruminococcaceae	*Ruminococcus*	*albus*	7	Bovine rumen, unknown^a^	Type strain	DSMZ
11	Streptococcaceae	*Streptococcus*	*equinus*	Pearl 11	Cow dung, unknown^a^		DSMZ
12	Streptococcaceae	*Streptococcus*	*gallolyticus*	DSM 16831	Koala feces, Australia	Type strain	DSMZ
13	Veillonellaceae	*Megasphaera*	*elsdenii*	B159	Cow rumen, USA		Dr. P. Weimer
14	Veillonellaceae	*Megasphaera*	*elsdenii*	T81	Cow rumen, USA		Dr. P. Weimer
15	Veillonellaceae	*Megasphaera*	*elsdenii*	LC1	Sheep rumen, unknown^a^	Type strain	DSMZ
16	Veillonellaceae	*Megasphaera*	*elsdenii*	2602A	Cow rumen, USA		Dr. P. Weimer
17	Veillonellaceae	*Megasphaera*	*elsdenii*	3016B	Cow rumen, USA		Dr. P. Weimer
18	Veillonellaceae	*Megasphaera*	*elsdenii*	3218A	Cow rumen, USA		Dr. P. Weimer
19	Veillonellaceae	*Megasphaera*	*elsdenii*	3436A	Cow rumen, USA		Dr. P. Weimer
20	Veillonellaceae	*Megasphaera*	*elsdenii*	4251	Cow rumen, USA		Dr. P. Weimer
21	Veillonellaceae	*Megasphaera*	*elsdenii*	4257	Cow rumen, USA		Dr. P. Weimer
22	Veillonellaceae	*Megasphaera*	*elsdenii*	4296	Cow rumen, USA		Dr. P. Weimer
23	Veillonellaceae	*Megasphaera*	*elsdenii*	4400	Cow rumen, USA		Dr. P. Weimer
24	Veillonellaceae	*Megasphaera*	*elsdenii*	5045	Cow rumen, USA		Dr. P. Weimer
25	Veillonellaceae	*Megasphaera*	*elsdenii*	5052B	Cow rumen, USA		Dr. P. Weimer
26	Veillonellaceae	*Megasphaera*	*elsdenii*	5596	Cow rumen, USA		Dr. P. Weimer
27	Veillonellaceae	*Selenomonas*	*ruminantium*	GA-192	Bovine rumen, USA	Subsp. *ruminantium*, type strain	DSMZ
28	Veillonellaceae	*Selenomonas*	*ruminantium*	PC 18	Bovine rumen, USA	Subsp. *lactilytica*, type strain	DSMZ

^a^ Unknown, country of origin unknown

Four different growth media were used: i/ control medium (pH = 6.70 ± 0.203; mean ± SD), ii/ lactate-enriched medium (pH = 6.82 ± 0.457; mean ± SD), iii/ low pH medium (pH = 5.50 ± 0.005; mean ± SD), and iv/ docosahexaenoic acid (DHA)-enriched medium (pH = 6.62 ± 0.150; mean ± SD). A slightly modified *Butyrivibrio* medium (medium 704; Deutsche Sammlung von Mikroorganismen and Zellkulturen GmbH, Braunschweig, Germany) was used as a control medium. The modification was based on Jeyanathan et al. [53]). The control medium used in the current set-up contained (per L): 90 mL mineral solution (per L distilled water, 6 g KH_2_PO_4_, 12 g NaCl, 6 g (NH_4_)_2_SO_4_, 1.6 g CaCl_2_.2H_2_O, 2.5 g MgSO_4_.7H_2_O), 150 mL rumen fluid, 0.3 g K_2_HPO_4_, 2 g trypticase peptone, 2 g yeast extract, 0.5 mL Na-resazurin solution, 4 g Na_2_CO_3_, 1 g each of glucose, maltose, cellobiose and starch, and 0. 5 g of L-Cysteine-HCl., Compared with the original basic medium, a volatile FA (VFA) mixture (3.1 mL/L), hemin (2 mL/L) and glycerol (0.5 g/L) were omitted from the basic medium (as these substrates are supposed to be present in the rumen fluid), and L-cysteine-HCl (0.5 g/L) was used as the only reducing agent whereas in the original basic medium a mixture (1:1) of L-Cysteine-HCl and Na_2_S.9H_2_O was used. Some further modifications were applied to the preparation of rumen fluid (see further) and the rumen fluid/buffer ratio (20% rumen fluid instead of 15%, v/v). The rumen fluid was collected from three adult sheep. These were fitted with a ruminal cannula and were fed grass hay ad libitum and a commercial, pelleted grain-based concentrate (200 g/d) twice a day, at 09 h00 and 17 h00, according to their maintenance requirements. Approximately 0.5 L of ruminal digesta was collected from each animal just before the morning feeding. The collected rumen fluid was filtered through a sieve with a pore size of 1 mm and combined. Then, the combined rumen fluid was sterilized by autoclaving for 20 min at 121 °C. To remove fine particles, the rumen fluid was centrifuged twice, the first time at 14,000×*g* and the second time at 20,000×*g*, each time for 15 min at 4 °C. The supernatant was stored at − 20 °C and thawed before use. The lactate-enriched medium was the control medium supplemented with Na-lactate (Sigma-Aldrich, Diegem, Belgium) to a final concentration of 200 mM (based on a growth medium specifically used for *M. elsdenii* in Weimer and Moen [42]). Initially, a lower concentration more closely related to potential rumen lactate concentrations (i.e. 9 mM) was used (data not shown), however, as no differences were observed in OD_600_, it was decided to use a higher concentration. The low pH medium was prepared by adding 2 M HCl solution to the control medium to reduce the pH from 6.5 to 5.5. The DHA-enriched medium was the control medium supplemented with DHA (Nu-Chek Prep, Elysian, MN, USA) to a final concentration of 40 μg/mL.

These media were transferred to Hungate-type tubes (16 mm i.d., 125 mm long; Chemglass Life Sciences, Vineland, NJ, USA). Then, 18:3*n*-3, 18:2*n*-6 or *trans*-11 18:1 (Nu-Chek Prep, Elysian, MN, USA) was added into each tube to a final concentration of 40 μg/mL, after which the tubes were closed with screw caps fitted with butyl rubber septa (Chemglass Life Sciences, Vineland, NJ, USA). The tubes were then autoclaved (121 °C, 20 min) prior to inoculum addition. All preparations and transfers were carried out under continuous flushing of CO_2_.

The inoculum was harvested from fresh cultures of each strain, grown in modified control medium (30% rumen fluid instead of 20%, v/v) for 12 to 36 h, depending on the growth rate of the strain (OD_600_ = 1.26 ± 0.41; mean ± SD). After inoculum addition, the tubes were maintained under anaerobic conditions at 39 °C, with intermittent shaking in a batch culture incubator (Edmund Bühler GmbH, Hechingen, Germany). The reactions were stopped at different time points (according to the experimental design) by removing the tubes from the incubator and cooling the tubes in an ice bath. Optical density at 600 nm (OD_600_; Ultraspec10, Amersham Biosciences Corp., Piscataway, NJ, USA) and pH (Hanna Instruments, Temse, Belgium) were measured, and subsamples were collected for analysis of VFA (2 mL) and long-chain FA (LCFA; remainder of the tube, i.e. 8 mL).

### Fatty acid solution

22:6*n*-3, 18:3*n*-3 and 18:2*n*-6 solutions were prepared as a watery FA solution containing either 10 g/L 18:3*n*-3 or 18:2*n*-6 (control medium, lactate-enriched medium and low pH medium) or 10 g/L 18:2*n*-6 and 10 g/L 22:6*n*-3 (DHA-enriched medium), together with 166.7 mL/L Tween 20 solution (75 g/L; Sigma-Aldrich, Diegem, Belgium) and 12.5 mL/L 3 M NaOH (based on Jeyanathan et al. [53]). Tween 20 was added as an emulsifier, whilst NaOH was added in order to obtain a clear solution. *Trans*-11 18:1 was dissolved in dimethyl sulfoxide to a final concentration of 10 g/L. The required amount of each FA solution was added individually to each Hungate tube before autoclaving. The amounts of Tween-80, NaOH and dimethyl sulfoxide were kept constant in all tubes .

### Experimental design

Table 7 summarizes the experimental setup of the current study. In experiment 1 (Exp. 1), pure cultures of the 28 strains were incubated individually under control growth conditions in the presence of 40 μg/mL 18:3*n*-3 or 18:2*n*-6 or *trans*-11 18:1 in order to determine their ability to metabolize 18:3*n*-3, 18:2*n*-6 and *trans*-11 18:1. The dose of the FA was decided based on the preliminary in house experiments performed with *B. fibrisolvens* and *C. acnes*. To obtain an inoculum size of 5% (v/v), 0.5 mL inoculum was added to 9.5 mL of growth medium. The incubations were stopped after 24 h to evaluate the disappearance of 18:3*n*-3, 18:2*n*-6 or *trans*-11 18:1 and the intermediates formed.

**Table 7:** Overview of the different pure culture experiments conducted

	Bacterium^a^ (inoculum size, v/v)	Ratio of *B. fibrisolvens* (%)/*C. acnes* (%) in the inoculum^b^	Growth medium^c^	Fatty acid substrate^d^	Incubation period (h)
Exp. 1	28 strains individually (Table 1) (5%)	NA	Control	18:3*n*-3	24
				18:2*n*-6	24
				*trans*-11 18:1	24
Exp. 2	28 strains individually (Table 1) (5%)	NA	Lactate	18:2*n*-6	24
Exp. 3	*B. fibrisolvens* (5%)	100/0	Control	18:2*n*-6	0, 2, 4, 8, and 24
			Low pH	18:2*n*-6	0, 2, 4, 8, and 24
			DHA	18:2*n*-6	0, 2, 4, 8, and 24
	*C. acnes* (5%)	0/100	Control	18:2*n*-6	0, 2, 4, 8, and 24
			Low pH	18:2*n*-6	0, 2, 4, 8, and 24
			DHA	18:2*n*-6	0, 2, 4, 8, and 24
Exp. 4	*B. fibrisolvens* (10%)	100/0	Control	18:2*n*-6	0, 4, 8, and 24
			Low pH	18:2*n*-6	24
			DHA	18:2*n*-6	24
	*B. fibrisolvens* (5%) and *C. acnes* (5%)	50/50	Control	18:2*n*-6	0, 4, 8, and 24
			Low pH	18:2*n*-6	24
			DHA	18:2*n*-6	24
	*B. fibrisolvens* (1%) and *C. acnes* (9%)	10/90	Control	18:2*n*-6	0, 4, 8, and 24
			Low pH	18:2*n*-6	24
			DHA	18:2*n*-6	24
	*B. fibrisolvens* (0.2%) and *C. acnes* (9.8%)	2/98	Control	18:2*n*-6	0, 4, 8, and 24
			Low pH	18:2*n*-6	24
			DHA	18:2*n*-6	24
	*B. fibrisolvens* (0.04%) and *C. acnes* (9.96%)	0.4/99.6	Control	18:2*n*-6	0, 4, 8, and 24
			Low pH	18:2*n*-6	24
			DHA	18:2*n*-6	24
	*C. acnes* (10%)	0/100	Control	18:2*n*-6	0, 4, 8, and 24
			Low pH	18:2*n*-6	24
			DHA	18:2*n*-6	24

^a^ Fresh cultures, grown in modified control medium (30% rumen fluid instead of 20%, v/v) for 12 to 36 h, depending on the growth rate of the strain (OD_600_ = 1.26 ± 0.41; mean ± SD), were used as inoculum

^b^ NA, not applicable

^c^ Lactate, control medium supplemented with 200 mM Na-lactate; low pH, control medium with pH adjusted to 5.5; DHA (docosahexaenoic acid), control medium containing 40 μg/mL of 22:6*n*-3

^d^ The initial amount of fatty acid was 40 μg/mL

In a second experiment (Exp. 2), the same 28 strains were incubated individually under lactate-enriched conditions in the presence of 40 μg/mL 18:2*n*-6 to assess the influence of lactate on 18:2*n*-6 metabolism. The inoculum size was 5% (v/v) and incubations were stopped after 24 h.

In experiment 3 (Exp. 3), pure cultures of *B. fibrisolvens*, as a *cis*-9, *trans*-11 CLA producer, and *C. acnes*, as a *trans*-10, *cis*-12 CLA producer, were grown separately in three different growth media (control, low pH, and DHA-enriched media) in the presence of 40 μg/mL 18:2*n*-6 to examine the effect of growth medium on the rate of CLA formation by these bacterial species. In order to obtain an inoculum size of 5% (v/v), 0.5 mL inoculum was added to 9.5 mL growth medium. The incubations were stopped at different time points (0, 2, 4, 8 and 24 h) to evaluate the effect of incubation time on CLA formation.

In experiment 4 (Exp. 4), pure cultures of *B. fibrisolvens* and *C. acnes* were combined to establish different biomass ratios of those species in order to elucidate their effect on the *trans*-11 to *trans*-10 shift, in the presence of 40 μg/mL 18:2*n*-6. Different volumes of the two inoculum sources were mixed together in order to obtain the different ratios. The total inoculum size was 10% (v/v). The incubations were stopped at different time points (0, 4, 8 and 24 h or 24 h only) depending on the growth medium.

For all experiments mentioned above, each treatment was performed in quadruplicate (analytical replicates). These quadruplicates were spread into duplicates, which were performed on different days. For each quadruplicate, the inoculum was grown separately. On each day, blank samples, containing distilled water as inoculum, were added in duplicate to measure the initial amount of VFA and LCFA before the incubation.

### In vitro incubation with mixed rumen inoculum (Exp. 5)

When 18:3*n*-3 or 18:2*n*-6 was converted to a hydroxy FA after 24 h of incubation by the pure culture in experiment 1, a follow-up incubation was performed to elucidate whether the formed intermediate is further converted to *trans*-10 intermediates by mixed rumen inoculum (Exp. 5). As sheep seem to be less vulnerable to situations associated with a *trans*-10 shift compared with cows (e.g. [54]), cow rumen inoculum was used as donor of rumen microbes for this in vitro incubation. The rumen fluid was collected from three lactating Holstein-Friesian dairy cows (analytical replicates), each fitted with a ruminal cannula. The cows were fed a basal diet consisting of maize silage and grass silage (50/50, w/w DM basis) ad libitum, supplemented with a standard concentrate according to their milk production. Samples of ruminal digesta were collected from each animal just before the morning feeding, after which it was filtered through a sieve with a pore size of 1 mm under continuous flushing with CO_2_. The filtered rumen fluid was mixed with a bicarbonate/phosphate buffer (rumen fluid/buffer, 1/4 v/v) containing 0.2125 g glucose, 0.2125 g cellobiose, 0.1875 g xylose, 0.1875 g arabinose, 1.25 g acid casein hydrolysate, 1.25 g peptone, 1.25 g yeast extract, 2.685 g Na_2_HPO_4_·12H_2_O, 1.1625 g KH_2_PO_4_, 0.093 g MgCl_2_·6H_2_O, 6.555 g NaHCO_3_ and 0.75 g NH_4_HCO_3_ per liter of distilled water (pH ≈ 6.8).

To assess further metabolism of hydroxy FA, these FA were produced in incubations with the bacterial strains identified during the first part of the study to biohydrate either 18:3*n*-3 or 18:2*n*-6 using the same methodology as described above (Exp. 1). Of each treatment (strain × 18:3*n*-3/18:2*n*-6), 10 Hungate tubes were incubated for 24 h starting from the same inoculum. Then, those tubes were pooled, 25 mL was transferred into 120-mL incubation flasks, after which it was freeze-dried. The amounts of hydroxy FA, accumulating at the end of the initial incubation were quantified for each bacterial strain. Into the flasks containing 25 mL of the freeze-dried pure culture medium with the hydroxy FA, a 25 mL rumen fluid/buffer mixture was added. The cultures were maintained under anaerobic conditions at 39 °C, with intermittent shaking in a batch culture incubator (Edmund Bühler GmbH, Hechingen, Germany). Reactions were stopped after 24 h of incubation by cooling in an ice bath, after which pH (Hanna Instruments, Temse, Belgium) was measured and subsamples were collected for analysis of VFA (2 mL) and LCFA (10 mL).

### Chemical analysis and calculations

For VFA analysis, 2 mL of incubation medium was collected and acidified immediately with 200 μL of formic acid, which contained an internal standard (10 mg of 2-ethylbutyric acid (Sigma-Aldrich, Diegem, Belgium) per mL of formic acid). After centrifugation (15 min at 4 °C and 31,000×*g*), the supernatant was filtered and transferred into a 1.5 mL glass vial. Samples were stored at 4 °C until analysis using a gas chromatograph (HP7890A; Agilent Technologies, Diegem, Belgium) equipped with a Supelco Nukol capillary column (30 m × 0.25 mm i.d. × 0.25 μm thickness; Sigma-Aldrich, Diegem, Belgium) and a flame ionization detector. The temperature program was as follows: 120 °C at injection for 0.2 min, increased at 10 °C/min to 180 °C, and kept at this temperature for 3 min; injector temperature: 250 °C; detector temperature: 255 °C. For this temperature program, 0.3 μL was injected with a split/split less ratio of 25:1 using H_2_ as carrier gas at 0.8 mL/min. Volatile FA peaks were identified based on their retention times, compared to external standards (Sigma-Aldrich, Diegem, Belgium). Net production of VFA was calculated by subtracting the amount in the incubation medium before incubation from the amount after incubation.

For analysis of LCFA, incubation medium (8 mL for pure culture incubations, Exp. 1–4; and 10 mL for the mixed community incubations, Exp. 5) was collected in glass tubes at 0 h and after the incubation, and immediately frozen at − 20 °C and freeze-dried prior to further analysis. Fatty acids were methylated as described by Dewanckele et al. [18]. Briefly, toluene (2 mL) containing the internal standard (21:0; Sigma-Aldrich, Diegem, Belgium) and methanolic NaOH (2 mL; 0.5 M) were added and the mixture was incubated at 70 °C for 60 min. This was followed by 30 min at 50 °C after addition of methanolic HCl (3 mL), prepared by dissolving acetyl chloride in methanol (5/1, v/v). Fatty acid methyl esters (FAME) were extracted with hexane. Analysis of FAME was carried out using a gas chromatograph (HP7890A; Agilent Technologies, Diegem, Belgium) equipped with an SP-2560 capillary column (75 m × 0.18 mm i.d. × 0.14 μm thickness; Supelco Analytical, Bellefonte, PA, USA) and a flame ionization detector. The temperature program was as follows: initially 70 °C for 2 min, increasing by 15 °C/min to 150 °C, followed by a second increase at 1 °C/min up to 165 °C and holding for 12 min, followed by a third increase at 2 °C/min to 170 °C, held at 170 °C for 5 min, increased at 5 °C/min to 215 °C, and held at 215 °C for 20 min. Inlet and detector temperatures were 250 °C and 255 °C, respectively. Injection volume was 1 μL with a split ratio of 25:1. Hydrogen was used as the carrier gas at a flow rate of 1 mL/min.

Peaks were routinely identified based on retention time comparisons with commercial mixtures of methyl ester standards (GLC463, *cis*-9, *trans*-11 CLA and *trans*-10, *cis*-12 CLA; Nu-Chek Prep, Elysian, MN, USA). Methyl esters of formed intermediates not contained in commercially available standards were identified by GC-MS analysis of 4,4-dimethyloxazoline (DMOX) derivatives, prepared from FAME by using a modified procedure [55]. Briefly, FAME were converted into DMOX derivatives with 250 mg 2-amino-2-methyl-1-propanol under a nitrogen atmosphere at 175 °C overnight. DMOX derivatives were extracted twice with diethyl ether/hexane (1/1, v/v) and sodium chloride saturated water. The organic layer was dried with anhydrous sodium sulphate for 1 h, followed by evaporation until dry under nitrogen. The DMOX derivatives were dissolved in hexane. Impact ionization spectra of DMOX derivatives were obtained using a gas chromatograph equipped with a quadrupole mass detector (Trace DSQ II, Thermo Scientific, Waltham, MA, USA). Mass spectra were recorded under an ionization voltage of 70 eV (EI+ mode), using Xcalibur software (version 1.4 SR1) for data acquisition and processing. The column was a SLB-IL 60 capillary column (30 m × 0.25 mm i.d. × 0.2 μm thickness; Sigma-Aldrich, Diegem, Belgium). The oven temperature program was as follows: initially 50 °C for 2 min, increasing by 5 °C/min to 210 °C and holding for 10 min, followed by a second increase at 5 °C/min to 280 °C and held at 280 °C for 5 min. Interface and ion source temperatures were both 250 °C and the mass-to-charge ratio interval was 50–650 a.m.u. at 5.0 scans per second. Injections were carried out in splitless mode and helium (1.2 mL/min) was used as carrier gas. The obtained electron impact ionization spectra were used to locate double bonds based on atomic mass unit distances with an interval of 12 a.m.u. between the most intense peaks of clusters of ions containing n and n-1 carbon atoms, being interpreted as cleavage of the double bond between carbon n and n + 1 in the FA moiety. Besides this, the spectra were also used to locate hydroxyl groups based on comparisons with other reports and with the LipidWeb (www.lipidhome.co.uk).

Quantification of LCFA was based on the area of the internal standard and on the conversion of peak areas to the weight of FA by a theoretical response factor for each FA [56, 57].

For experiment 3 and 4, for each of the formed intermediates (i.e. *cis*-9, *trans*-11 CLA, *trans*-10, *cis*-12 CLA, *trans*-11 18:1 and *trans*-10 18:1) and the initial product (i.e. 18:2*n*-6), the proportion of total intermediates and initial product was calculated at each sampling time. This proportion was presented in relation to incubation time (x-axis). From this, the area under the curve was calculated over the 24 h of incubation (area units: % of total intermediates and 18:2n-6 × hour). The average proportion over this 24 h incubation period was then calculated as the area under the curve divided by 24. The average proportion should not be confused with the proportion after 12 h of incubation (= after half of the incubation period). In experiment 3, this calculation was done for 18:2*n*-6 and its biohydrogenation intermediates for each treatment combination (bacterium × growth medium) to examine the effect of bacterial species and growth medium on the metabolism of 18:2*n*-6. In experiment 4, the same calculation was done, but only for the control medium, to examine the effect of the biomass ratio of *B. fibrisolvens* to *C. acnes* in the inoculum on the metabolism of 18:2*n*-6. To elucidate the influence of growth medium, the individual measured values for each FA after 24 h of incubation were used.

### Identification of unknown fatty acids by GC/MS

**Fig. 4:**
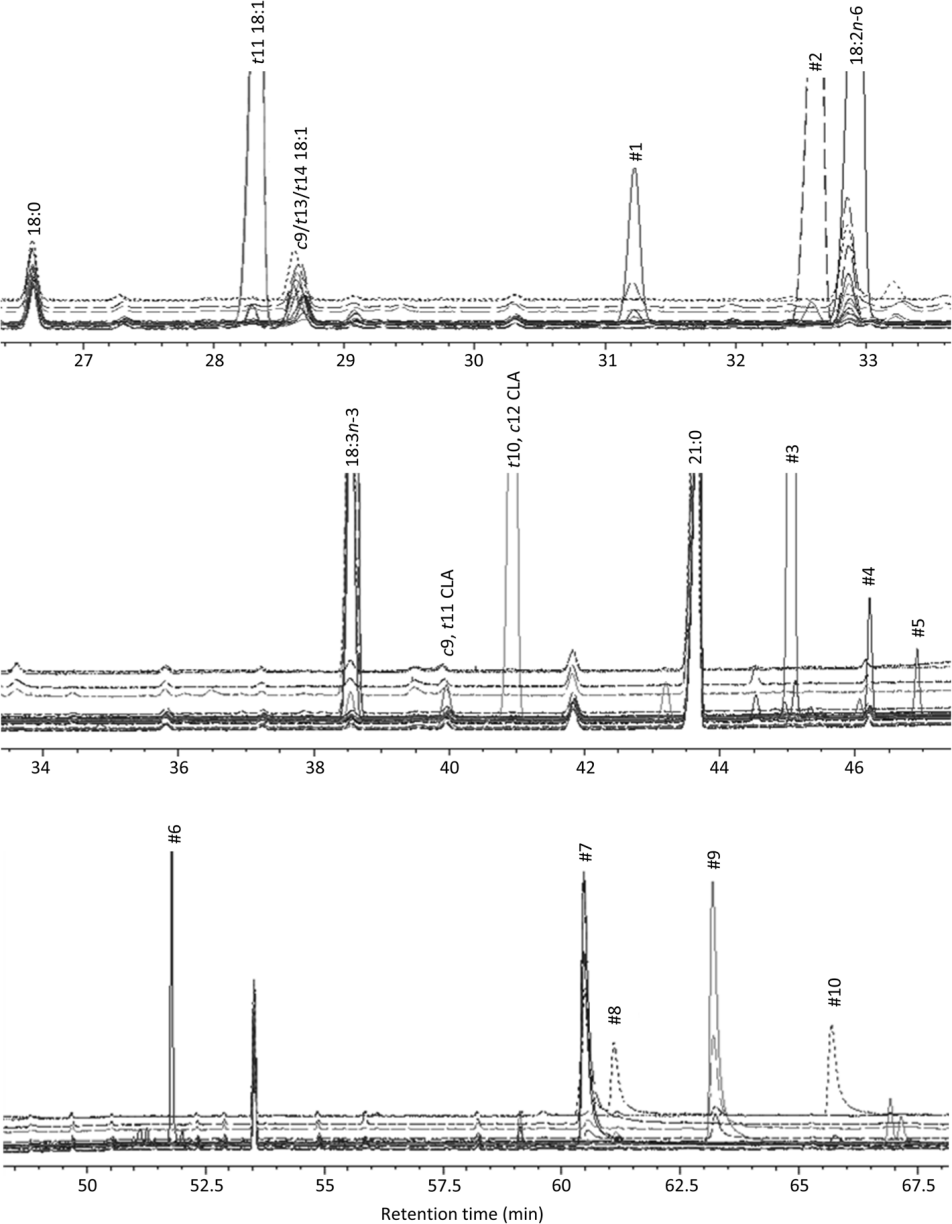
Partial GC-MS chromatogram of the C18 FAME elution profile region. *t*, *trans*; *c*, *cis*; CLA, conjugated linoleic acid; #1-#10, unidentified compounds. Different chromatograms originate from incubations with distilled water or with pure cultures of *Butyrivibrio fibrisolvens* D1, *Cutibacterium acnes* DSM 1897, *Streptococcus gallolyticus* DSM 16831, *Streptococcus equinus* Pearl 11, *Megasphaera elsdenii* 2602A and 5052B under control growth conditions

**Fig. 5:**
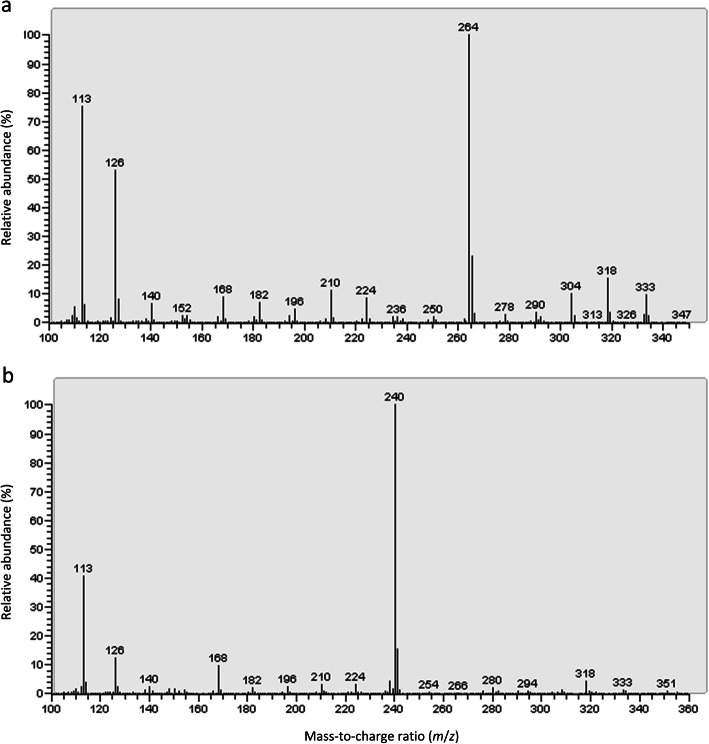
Examples of electron impact mass spectra of DMOX derivatives. **a**, compound #2, derived from pure culture incubation of *Butyrivibrio fibrisolvens* D1 under control growth conditions with 40 μg/mL 18:3*n*-3; **b**, compound #8, derived from pure culture incubation of *Megasphaera elsdenii* 5052B under control growth conditions with 40 μg/mL 18:2*n*-6

DMOX derivatives were prepared from the FAME extracts in order to characterize unknown biohydrogenation intermediates by electron impact mass spectrometry. In total, 10 isomers were characterized by GC/MS (Fig. 4). The obtained mass spectra were compared with mass spectra from intermediates of 18:3*n*-3 or 18:2*n*-6 biohydrogenation studies described in other reports (e.g. [29, 37, 38]) and with information from the LipidWeb (www.lipidhome.co.uk). In each mass spectrum with unknown isomers (Fig. 5), the base peak was observed at *m*/*z* 113, produced by a McLafferty arrangement. This peak was accompanied with a prominent ion at *m*/*z* 126, which is a characteristic ion in a DMOX derivative, and is formed by a cyclization displacement reaction.

The mass spectra of compound #1 showed a gap of 12 a.m.u. at *m*/*z* 196/208 and at *m*/*z* 250/262. Therefore, this peak was identified as ∆9,14–18:2. The double bond geometry could not be assigned through the used technique. The mass spectra of compound #2 corresponded to the mass spectra of ∆11,15–18:2 obtained in the experiment of Alves and Bessa [38], i.e. there was a gap of 12 a.m.u. at *m*/*z* 224/236 and at *m*/*z* 278/290. Since this isomer was produced when 18:3*n*-3 was incubated with *B. fibrisolvens* D1, which is an important *trans*-11 producer [6], we feel confident that the structure of ∆11,15–18:2 is *trans*-11, *cis*-15 18:2, in accordance with Alves and Bessa [38]. Compounds #3, #4, #5 and #6 were identified in a similar way as *cis*-9, *trans*-11, *cis*-15 CLnA, *trans*-10, *cis*-12, *cis*-15 CLnA, ∆9,11,15–18:3 and ∆11,13,15–18:3, respectively [37].

Compound #7 was identified as 13-OH ∆9–18:1. The double bond was recognized by the gap of 12 a.m.u. at *m*/*z* 196/208, whereas the ions at *m*/*z* 250 and *m/z* 280 indicate the presence of a hydroxyl group at carbon atom 13. Compounds #8, #9 and #10 were identified in a similar way as 10-OH ∆12–18:1, 13-OH ∆9,15–18:2 and 10-OH ∆12,15–18:2, respectively.

### Statistical analysis

Data were analyzed using the MIXED procedure of SAS (version Enterprise Guide 7.1; SAS Institute Inc., Cary, NC, US). In experiment 2, the following model was used: *Y*_*ij*_ *= μ* + *M*_*i*_ + *D*_*j*_ + *Ɛ*_*ij*_, with *M*_*i*_ the fixed effect of growth medium (*i* = control or lactate-enriched medium), *D*_*j*_ the random effect of day, and *Ɛ*_*ij*_ the residual error term. In experiment 3 and 4, the following model was used: *Y*_*ijk*_ = *μ* + *B*_*i*_ + *M*_*j*_ + *D*_*k*_ + *B*_*i*_ × *M*_*j*_ + *Ɛ*_*ijk*_, with *B*_*i*_ the fixed effect of bacterial species (exp. 3, *i* = *B. fibrisolvens* or *C. acnes*), or the fixed effect of the biomass ratio of *B. fibrisolvens* to *C. acnes* in the inoculum (exp. 4, *i* = 100/0, 50/50, 10/90, 2/98, 0.4/99.6, or 0/100), *M*_*j*_ the fixed effect of growth medium (*j* = control, low pH, or DHA-enriched medium), *D*_*k*_ the random effect of day, and *Ɛ*_*ijk*_ the residual error term. Differences at *P* <  0.05 were considered to be statistically significant and were evaluated by the Tukey-Kramer multiple comparison test.

## Supplementary information

**Additional file 1: Table S1.** Influence of lactate on OD_600_ of different bacterial strains after 24 h of incubation with 40 μg/mL 18:2*n*-6 (Exp. 2). **Table S2.** Influence of lactate on volatile fatty acids produced by different bacterial strains after 24 h of incubation with 40 μg/mL 18:2*n*-6 (exp. 2). **Table S3.** Net production (μmol per tube) of propionic acid and butyric acid by different biomass ratios of *Butyrivibrio fibrisolvens* D1 to *Cutibacterium acnes* DSM 1897 in the inoculum after 24 h of incubation under different growth conditions^a^.

## Data Availability

The datasets used and/or analyzed during the current study are available from the corresponding author on reasonable request.

## Abbreviations

CLA: Conjugated linoleic acid
CLnA: Conjugated linolenic acid
DHA: Docosahexaenic acid, 22:6*n*-3
DMOX: 4,4-dimethyloxazoline
FA: Fatty acid
FAME: Fatty acid methyl ester
LCFA: Long-chain fatty acid
OD: Optical density
VFA: Volatile fatty acid
