# Supplementary material for: Identifying and exploring biohydrogenating rumen bacteria with emphasis on pathways including trans-10 intermediates
Source: BMC Microbiol. 2020 Jul 7;20:198. doi: 10.1186/s12866-020-01876-7 (PMC7339423; doi:10.1186/s12866-020-01876-7)
Supplement: Supplementary file 1 — Additional file 1: Table S1. Influence of lactate on OD600 of different bacterial strains after 24 h of incubation with 40 μg/mL 18:2n-6 (Exp. 2). Table S2. Influence of lactate on volatile fatty acids produced by different bacterial strains after 24 h of incubation with 40 μg/mL 18:2n-6 (exp. 2). Table S3. Net production (μmol per tube) of propionic acid and butyric acid by different biomass ratios of Butyrivibrio fibrisolvens D1 to Cutibacterium acnes DSM 1897 in the inoculum after 24 h of incubation under different growth conditionsa. [file 12866_2020_1876_MOESM1_ESM.docx]

**SUPPLEMENTARY MATERIAL: Identifying and exploring biohydrogenating rumen bacteria with emphasis on pathways including *trans*-10 intermediates**

Lore Dewanckele,* Jeyamalar Jeyanathan,* Bruno Vlaeminck,† Veerle Fievez‡

Laboratory for Animal Nutrition and Animal Product Quality (Lanupro), Department of Animal Sciences and Aquatic Ecology, Ghent University, Ghent, Belgium

*Shared first authorship

†Present address: Research Group Marine Biology, Department of Biology, Ghent University, Ghent, Belgium.

‡Address correspondence to Veerle Fievez, Veerle.Fievez@UGent.be.

**Table S1** Influence of lactate on OD_600_ of different bacterial strains after 24 h of incubation with 40 µg/mL 18:2*n*-6 (Exp. 2)

| Strain | Control | Lactate | SEM | *P*-value |
| --- | --- | --- | --- | --- |
| *Acidaminococcus fermentans* VR4 | 0.15 | 0.06 | 0.015 | 0.003 |
| *Acidaminococcus intestini* ADV 255.99 | 0.24 | 0.08 | 0.050 | < 0.001 |
| *Bifidobacterium adolescentis* RU 424 | 0.47 | 0.28 | 0.131 | 0.340 |
| *Bifidobacterium pseudolongum* RU224 | 0.44 | 0.38 | 0.143 | 0.761 |
| *Butyrivibrio fibrisolvens* D1 | 1.17 | 1.17 | 0.094 | 0.986 |
| *Butyrivibrio proteoclasticus* P18 | 1.11 | 1.07 | 0.047 | 0.577 |
| *Lactobacillus ruminis* RF1 | 1.04 | 0.86 | 0.019 | 0.001 |
| *Lactobacillus ruminis* RF2 | 1.00 | 0.82 | 0.019 | < 0.001 |
| *Cutibacterium acnes* DSM 1897 | 1.17 | 0.67 | 0.285 | 0.281 |
| *Ruminococcus albus* 7 | 0.06 | < 0.01 | 0.002 | < 0.001 |
| *Streptococcus equinus* Pearl 11 | 1.42 | 1.18 | 0.052 | 0.028 |
| *Streptococcus gallolyticus* DSM 16831 | 1.13 | 0.82 | 0.051 | 0.013 |
| *Megasphaera elsdenii* B159 | 1.19 | 1.40 | 0.082 | 0.157 |
| *Megasphaera elsdenii* T81 | 1.03 | 1.22 | 0.133 | 0.380 |
| *Megasphaera elsdenii* LC1 | 0.86 | 1.37 | 0.128 | 0.048 |
| *Megasphaera elsdenii* 2602A | 1.52 | 1.58 | 0.021 | 0.082 |
| *Megasphaera elsdenii* 3016B | 1.35 | 1.65 | 0.072 | 0.039 |
| *Megasphaera elsdenii* 3218A | 1.23 | 1.57 | 0.044 | 0.005 |
| *Megasphaera elsdenii* 3436A | 1.24 | 1.63 | 0.030 | < 0.001 |
| *Megasphaera elsdenii* 4251 | 1.25 | 1.56 | 0.088 | 0.065 |
| *Megasphaera elsdenii* 4257 | 1.31 | 1.65 | 0.051 | 0.009 |
| *Megasphaera elsdenii* 4296 | 1.29 | 1.70 | 0.050 | 0.005 |
| *Megasphaera elsdenii* 4400 | 0.13 | 0.12 | 0.085 | 0.953 |
| *Megasphaera elsdenii* 5045 | 1.34 | 1.58 | 0.033 | 0.006 |
| *Megasphaera elsdenii* 5052B | 1.39 | 1.20 | 0.032 | 0.013 |
| *Megasphaera elsdenii* 5596 | 1.29 | 1.55 | 0.037 | 0.008 |
| *Selenomonas ruminantium* GA-192 | 0.97 | 1.21 | 0.074 | 0.084 |
| *Selenomonas ruminantium* PC 18 | 0.92 | 1.82 | 0.062 | < 0.001 |

**Table S2** Influence of lactate on volatile fatty acids produced by different bacterial strains after 24 h of incubation with 40 µg/mL 18:2*n*-6 (exp. 2)

| Strain | Control | | Lactate | | SEM | *P*-value |
| --- | --- | --- | --- | --- | --- | --- |
|  | Total VFA formed (µmol/tube)^a^ | Main VFA products^b^ | Total VFA formed (µmol/tube)^a^ | Main VFA products^b^ |  |  |
| *Acidaminococcus fermentans* VR4 | 54.8 | A, B | 54.0 | A, B | 5.289 | 0.920 |
| *Acidaminococcus intestini* ADV 255.99 | 57.3 | A, B | 71.7 | A, B | 7.161 | 0.093 |
| *Bifidobacterium adolescentis* RU 424 | 249.7 | A | 235.7 | A | 9.774 | 0.155 |
| *Bifidobacterium pseudolongum* RU224 | 212.9 | A | 199.7 | A | 23.02 | 0.437 |
| *Butyrivibrio fibrisolvens* D1 | 128.4 | B, A | 146.6 | B, A | 6.398 | 0.115 |
| *Butyrivibrio proteoclasticus* P18 | 168.6 | B, A | 158.1 | B, A | 6.695 | 0.315 |
| *Lactobacillus ruminis* RF1 | 22.7 | A, P | 19.2 | A, B | 12.97 | 0.478 |
| *Lactobacillus ruminis* RF2 | 12.7 | A, P | 27.2 | A | 4.485 | 0.072 |
| *Cutibacterium acnes* DSM 1897 | 118.5 | P, A | 107.0 | P, A | 38.88 | 0.844 |
| *Ruminococcus albus* 7 | 29.8 | A | 18.2 | A | 3.121 | 0.047 |
| *Streptococcus equinus* Pearl 11 | 19.5 | A | 15.0 | A | 2.240 | 0.228 |
| *Streptococcus gallolyticus* DSM 16831 | 12.6 | A | 22.7 | A | 5.726 | 0.281 |
| *Megasphaera elsdenii* B159 | 149.2 | B | 913.1 | P, A, B | 16.37 | < 0.001 |
| *Megasphaera elsdenii* T81 | 125.7 | B | 822.9 | P, A, B | 40.47 | < 0.001 |
| *Megasphaera elsdenii* LC1 | 122.9 | B, A | 905.3 | A, P, B | 35.35 | < 0.001 |
| *Megasphaera elsdenii* 2602A | 191.2 | B, P | 1103.6 | P, A, B | 61.68 | < 0.001 |
| *Megasphaera elsdenii* 3016B | 136.6 | B | 885.7 | P, A, B | 27.70 | < 0.001 |
| *Megasphaera elsdenii* 3218A | 136.0 | B | 856.2 | P, A, B | 37.37 | < 0.001 |
| *Megasphaera elsdenii* 3436A | 117.1 | B | 861.7 | P. A, B | 23.09 | < 0.001 |
| *Megasphaera elsdenii* 4251 | 125.7 | B | 857.0 | P, A, B | 35.99 | < 0.001 |
| *Megasphaera elsdenii* 4257 | 125.7 | B | 851.6 | P, A, B | 32.36 | < 0.001 |
| *Megasphaera elsdenii* 4296 | 121.6 | B | 870.6 | P, A, B | 40.50 | < 0.001 |
| *Megasphaera elsdenii* 4400 | 59.0 | A, P | 80.3 | A, P | 37.34 | 0.707 |
| *Megasphaera elsdenii* 5045 | 126.7 | B | 803.1 | P, A, B | 51.76 | < 0.001 |
| *Megasphaera elsdenii* 5052B | 64.3 | A | 66.2 | A | 6.744 | 0.846 |
| *Megasphaera elsdenii* 5596 | 125.5 | B | 858.0 | P, A, B | 34.20 | < 0.001 |
| *Selenomonas ruminantium* GA-192 | 81.4 | P, A | 194.2 | P, A | 17.49 | 0.010 |
| *Selenomonas ruminantium* PC 18 | 241.9 | P, A | 1181.7 | P, A | 108.8 | 0.004 |

^a^ Measured fermentation products were acetate, propionate, isobutyrate, butyrate, isovalerate, valerate and caproate.

^b^ Main VFA product. A, acetate; B, butyrate; P, propionate; in decreasing order of importance.

**Table S3** Net production (µmol per tube) of propionic acid and butyric acid by different biomass ratios of *Butyrivibrio fibrisolvens* D1 to *Cutibacterium acnes* DSM 1897 in the inoculum after 24 h of incubation under different growth conditions^a^

|  |  | *B. fibrisolvens* (%)/*C. acnes* (%) | | | | | | SEM | *P*-value | |
| --- | --- | --- | --- | --- | --- | --- | --- | --- | --- | --- |
|  |  | 100/0 | 50/50 | 10/90 | 2/98 | 0.4/99.6 | 0/100 |  | Linear | Quadratic |
| Propionic acid | Control | 0.77 | 21.74 | 28.75 | 32.46 | 31.78 | 37.88 | 12.310 | < 0.001 | 0.832 |
|  | Low pH | 1.06* | 10.15* | 18.87* | 23.16* | 21.53* | 21.27* |  | < 0.001 | 0.698 |
|  | DHA | 1.24* | 15.48* | 25.49* | 28.06* | 26.97* | 28.65* |  | < 0.001 | 0.377 |
| Butyric acid | Control | 106.70 | 74.48 | 58.31 | 36.37 | 52.67 | 0.79 | 9.630 | 0.022 | 0.077 |
|  | Low pH | 8.69* | 4.24* | 1.82* | 1.23* | 0.69* | 0.50 |  | 0.359 | 0.967 |
|  | DHA | 20.81* | 26.10* | 15.11* | 4.91* | 3.20* | 0.69 |  | < 0.001 | 0.203 |

^a^ Low pH, control medium with pH adjusted to 5.5; DHA (docosahexaenoic acid), control medium containing 40 µg/mL of 22:6*n*-3; All growth media contained 40 µg/mL of 18:2*n*-6.

* Means differ (*P* < 0.05) from the control growth medium within the same ratio.
